# Rational design of hydrogels for immunomodulation

**DOI:** 10.1093/rb/rbac009

**Published:** 2022-02-22

**Authors:** Wenhuan Bu, Yuanhao Wu, Amir M Ghaemmaghami, Hongchen Sun, Alvaro Mata

**Affiliations:** 1 Liaoning Provincial Key Laboratory of Oral Diseases, School of Stomatology, China Medical University, No. 117, Nanjing North Street, Heping District, Shenyang 110001, China; 2 Department of Dental Materials, School of Stomatology, China Medical University, No. 117, Nanjing North Street, Heping District, Shenyang 110001, China; 3 Department of Center Laboratory, School of Stomatology, China Medical University, No. 117, Nanjing North Street, Heping District, Shenyang 110001, China; 4 School of Pharmacy, University of Nottingham, University Park, Nottingham NG7 2RD, UK; 5 Biodiscovery Institute, University of Nottingham, University Park, Nottingham NG7 2RD, UK; 6 Division of Immunology, School of Life Sciences, University of Nottingham, University Park, Nottingham NG7 2RD, UK; 7 Terasaki Institute for Biomedical Innovation, 1018 Westwood Blvd, Los Angeles, CA 90024, USA; 8 Department of Chemical and Environmental Engineering, University of Nottingham, University Park, Nottingham NG7 2RD, UK

**Keywords:** hydrogels, immunomodulation, physical properties, chemical properties

## Abstract

The immune system protects organisms against endogenous and exogenous harm and plays a key role in tissue development, repair and regeneration. Traditional immunomodulatory biologics exhibit limitations including degradation by enzymes, short half-life and lack of targeting ability. Encapsulating or binding these biologics within biomaterials is an effective way to address these problems. Hydrogels are promising immunomodulatory materials because of their prominent biocompatibility, tuneability and versatility. However, to take advantage of these opportunities and optimize material performance, it is important to more specifically elucidate, and leverage on, how hydrogels affect and control the immune response. Here, we summarize how key physical and chemical properties of hydrogels affect the immune response. We first provide an overview of underlying steps of the host immune response upon exposure to biomaterials. Then, we discuss recent advances in immunomodulatory strategies where hydrogels play a key role through (i) physical properties including dimensionality, stiffness, porosity and topography; (ii) chemical properties including wettability, electric property and molecular presentation;and (iii) the delivery of bioactive molecules via chemical or physical cues. Thus, this review aims to build a conceptual and practical toolkit for the design of immune-instructive hydrogels capable of modulating the host immune response.

## Graphical Abstract

**Figure:**
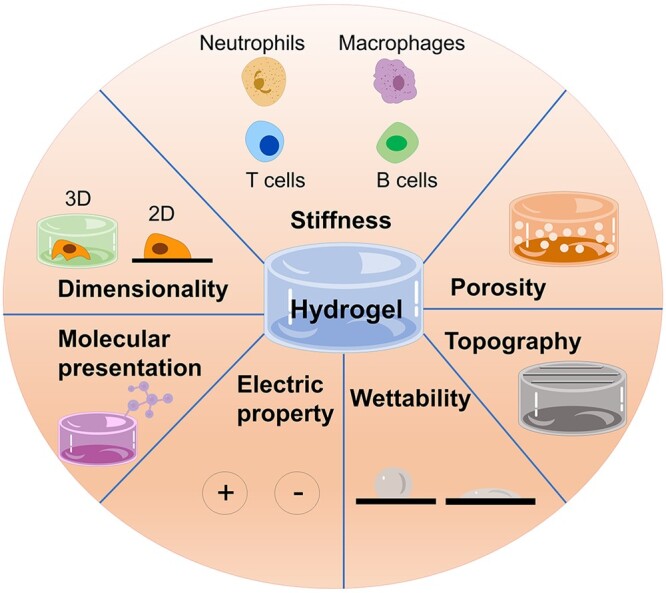

## Introduction 

### The immune system and immunomodulation

The immune system is the main line of defence that protects organisms against external challenges, such as infection caused by pathogens and internal ones, such as malignant cell phenotypes as a result of gene mutations. In such situations, the immune system is able to quickly respond to environmental stimuli to maintain immune homeostasis, which is critical for tissue development, repair and regeneration [1–3].

Diverse factors, such as age, genetics, infections and lifestyle can bring about hypoactive or hyperactive immune activity. These exaggerated immune responses often serve as the basis of immune-related diseases or even acute inflammatory reactions. In order to restore immune homeostasis to enhance tissue regeneration or promote healing post-tissue damage, there is increasing interest to find ways to control the immune response [4]. The main objective of immunomodulation is to either increase immune activity in hypoactive immune conditions, such as cancer and chronic infection, or decrease immune activity to withstand hyperactive immune response in inflammatory diseases including autoimmune disorders, allergy and transplant rejection. To achieve this, a large number of immunomodulatory biologics have been developed, including cytokines, antibodies, peptides, nucleic acids and drugs [5–7]. These biologics, however, are usually detrimental in systemic administration, enzymatically degraded, have short half-life, diffuse in body fluids leading to diluted action concentration and lack targeting ability. These weaknesses lead to increased dosage and frequency of use, posing a series of unwanted adverse reactions including cytokine release syndrome, immunogenicity, infections, hypersensitivity and malignancy [5, 8].

### Hydrogels for immunomodulation

The efficiency and biocompatibility of immunomodulatory biologics can be significantly improved through binding to or encapsulation within biomaterials. This approach provides protection from enzymatic degradation, excessive available concentration in target tissue and reduced adverse reactions. For example, when covalently bound to poly(amidoamine) dendrimer nanoparticles, methotrexate exhibited increased anti-inflammatory effects [9]. In addition, liposomal encapsulation enhanced prednisolone therapeutic potency to treat autoimmune encephalomyelitis, evidenced by the need for reduced dosages and application frequency [10]. Nevertheless, systematic administration of these nanomaterials, with or without target moieties, cannot guarantee their arrival to intended site, and locally injected nanomaterials may rapidly drain away from target site due to their small size. Macro-scale materials can address these issues. Up to now, many macro-scale biomaterials, including implantable and injectable biomaterials, have been designed to deliver immunomodulatory biologics for cancer immunotherapy and tissue repair [11, 12]. Among them, hydrogels, highly hydrated 3D cross-linked polymer or colloidal networks, are increasingly attractive biomaterials for a variety of biomedical applications. Given their high water content to emulate hydrated physiological environments and their versatility to be responsive, injected and biodegraded, hydrogels are promising candidates for designing immunomodulatory materials [13, 14]. As carriers, hydrogels loaded with immunomodulatory biologics could effectively regulate immune responses to promote tissue regeneration, prevent tumour growth and ameliorate immune-related pathologies [15–17]. However, there has been increasing interest to use cargo-free hydrogels as immunomodulators [18, 19], which are generating promising results [20, 21]. Nonetheless, to rationally design hydrogels with tunable immunomodulatory properties, it is critical to investigate and dissect confounding factors implicated in these studies, such as dimensionality, stiffness and topography.

It is well documented that cell response can be modulated through the microenvironmental cues presented by biomaterials [22, 23]. Hydrogels are particularly well positioned to be molecularly designed to signal cells through specific physical [24] and chemical [25] properties and their combination [26, 27] to control a variety of cell behaviours, such as adhesion, migration, proliferation and differentiation [28–31]. This opportunity is driving advances in hydrogel design and growing interest to use them not only to control cell growth [32, 33] but also to coordinate the behaviour of multiple cell types towards the generation of more complex spheroids [34, 35] and organoids [36]. However, their use to systematically investigate the effect of these properties on the behaviour of immune cells has been less explored, which represents an exciting opportunity to design immune-instructive hydrogels that can selectively enhance or reduce immune activity [37, 38].

As aforementioned, hydrogels are versatile materials enabling fine tuneability of their properties. Physical properties of hydrogels alter with the change of synthesis conditions. As a kind of 3D (dimensionality) scaffold, hydrogels provide cells with a simplified version of natural tissue environment in favour of cell survival [39]. Through regulating the concentration and type of cross-linker, hydrogels with different elasticity modulus (stiffness) and pore sizes (porosity) are synthesized [40]. Taking advantage of special templates, different patterns (topography) in micro- or nano-scale on the surface of the hydrogels are acquired [41–43]. Besides these physical properties, chemical properties are of equal importance and provide another opportunity to design and optimize hydrogels. Through grafting with relevant molecules, hydrogels can become hydrophilic or hydrophobic (wettability) [44]. In addition, hydrogels generally bear charges (electric property), which can be regulated by surface modification [45]. Moreover, distinct bioactive groups and molecules (molecular presentation) presented by hydrogels depending on their components, may affect cell behaviours [46]. Therefore, optimizing these properties and understanding their relationship with the immune response will contribute to developing immune-instructive hydrogels for biomedical application.

### Objective of the review

**Figure 1.**
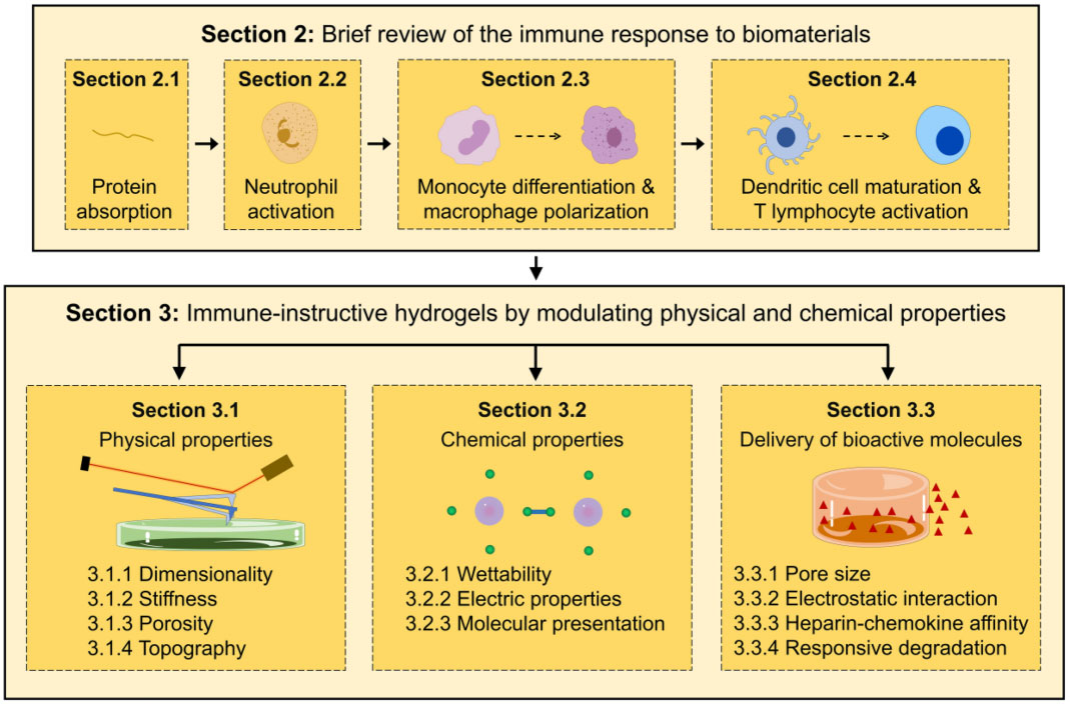
Structure of the topics presented and discussed in the review

Here, we aim to highlight current progress on the design of hydrogel materials for immunomodulation with a particular focus on the role of physical and chemical properties. For review articles focused on specific biological applications, the reader is referred to excellent reports by Singh *et al.* [6] and Wei *et al*. [14]. The review is divided in four sections (Fig. 1). First, a brief description of underlying events of the host immune response to implanted biomaterials is presented. Then, the main body of the review focuses on immunomodulatory strategies based on: (i) regulation of physical properties of hydrogels including dimensionality, stiffness, porosity and topography; (ii) modulation of chemical properties of hydrogels including wettability, electric property and molecular presentation; and (iii) delivery of bioactive molecules via physical or chemical cues. In this manner, we aim to provide an overview of how hydrogels interact with the immune system and build a practical toolbox for immune-informed biomaterials design.

## Brief review of the immune response to biomaterials

**Figure 2.**
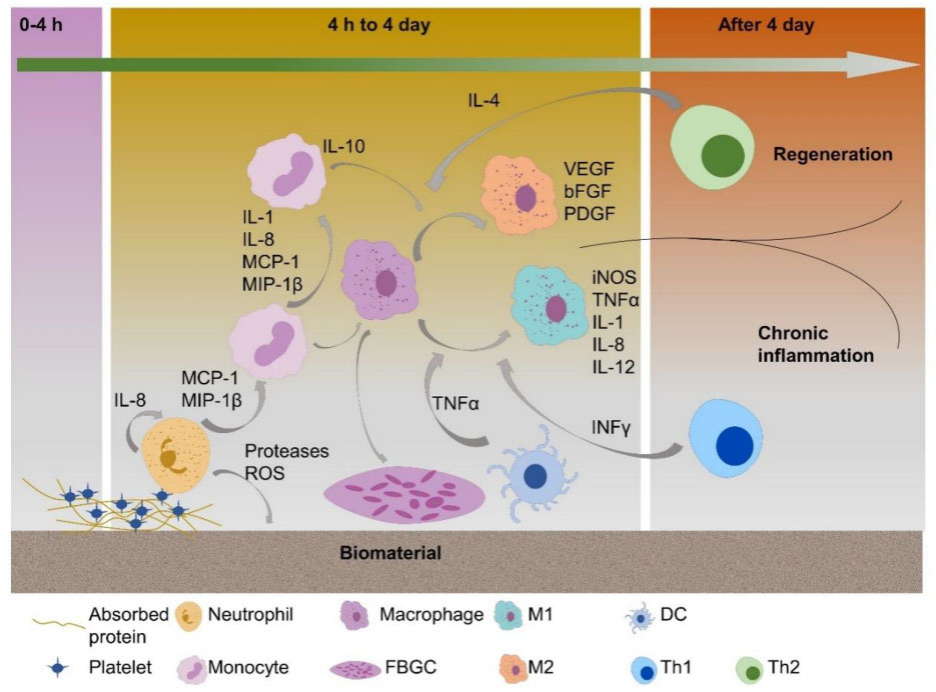
Illustration of the basic stages of the immune response to biomaterials

The immune system is composed of innate and adaptive systems that unceasingly surveil the organism to detect tissue insult, pathogen invasion or foreign bodies. A number of humoral and cellular factors from both types of immune systems including neutrophils, macrophages, dendritic cells (DCs) and lymphocytes, as well as cytokines secreted by these cells, are implicated in delivering an effective immune response. These factors interact with biomaterials spatio-temporally, triggering foreign-body reaction (FBR) in which a cascade of inflammation events give rise to fibrosis, cellular and collagenous deposition that encapsulates the implant [47]. Depending on the type of factors involved in the host response, the ultimate result of the FBR can be chronic inflammation or wound healing [38]. Here, we will briefly summarize the current understanding of immune events taking place as a result of biomaterial implantation (Fig. 2).

### Protein absorption initiates the immune response to biomaterials

Upon any kind of biomaterial implantation, an immune response is activated. Proteins from blood and interstitial fluids can non-specifically absorb to the biomaterial surface within nanoseconds after implantation [48]. In this scenario, biomaterial properties, such as surface chemistry, play a key role in defining the types and quantities of absorbed proteins. For example, Factor XII, the initiator of the coagulation cascade, is activated by contact with negatively charged substrates, which results in the release of thrombin [49]. This thrombin release can activate platelets that in turn release mediators of the coagulation pathway including prothrombinases that assemble and become activated on the surface of activated platelets [50, 51]. Then, thrombin formation arises, leading to further activation of platelets and coagulation factors, which amplify the coagulation cascade [51]. Besides thrombin, absorbed fibrinogen and tissue factors as well as complement also serves as adhesion substrate for platelets and induce activation of the attached platelets, which urge the formation of fibrin-rich clot [52–54]. As the depot of cytokines and growth factors, the clot serves as transient provisional matrix for cell adhesion and migration, and provides signals for initiating inflammation and wound healing [55].

### Neutrophil activation by biomaterials

Following protein absorption, neutrophils are recruited at the implantation site within hours and dominate the immune response for the first 2 days [56]. The neutrophil phagocytic response and degranulation are triggered upon protein-biomaterial contact, in which they secret a series of proteolytic enzymes and reactive oxygen species (ROS) with pathogen killing effect [57, 58]. These catalytic molecules and radicals may also damage the biomaterial as investigated in hydrogel degraded by elastase [59]. Simultaneously, neutrophil extracellular traps (NETs), a ‘sticky network’ of granular protein, elastase, chromatin DNA and histones from neutrophils, usually result in overproduction of a dense fibrotic matrix [60–62]. The excessive release of NETs will prevent integration between tissue and biomaterial, restraining the wound-healing process [62, 63]. Furthermore, neutrophils also release various cytokines to regulate host response, such as interleukin-8 (IL-8), activating neutrophils themselves, MCP-1 and MIP-1β, potent chemoattractants and activation factors for monocytes, macrophages, DC and lymphocytes [64].

### Monocyte differentiation and macrophage polarization by biomaterials

After neutrophil apoptosis, monocytes become the predominant cell type in the immune response. By secreting IL-1, IL-8, MCP-1, and MIP-1β, more monocytes are recruited at the implantation site and subjected to phenotypic conversion towards macrophages [64, 65]. These cells play an essential role in inflammation and tissue regeneration by releasing inflammatory mediators, tissue reorganization-related enzymes (e.g. metalloproteases) and fibroblast migration and proliferation-related cytokines and growth factors [66]. According to differences in their functions, macrophages are typically classified into two categories, referred to as classically activated (M1) and alternatively activated (M2) macrophages [67]. The term ‘macrophage polarization’ is used to describe this specific differentiation modality. M1 polarization is induced by stimulation of interferon-γ (IFNγ) from natural killer cells or T helper 1 (Th1) cells and TNFα from DCs, causing secretion of inflammatory cytokines such as induced nitric oxide synthase (iNOS), TNFα and IL-12 [68, 69]. These cytokines facilitate inflammation and recruit lymphocytes implicated in adaptive immunity [68]. On the other hand, IL-4 generated by granulocytes and Th2 cells as well as IL-10 generated by monocytes trigger M2 polarization [68, 70]. These M2 macrophages initiate down-regulation of inflammation, promotion of wound healing and generation of new blood vessels through deposition of extracellular matrix (ECM) and cell proliferation [68, 70]. However, sustained activation of both M1 and M2 macrophages could trigger tissue damage or fibrosis, respectively [71]. In addition, macrophages also affect immune responses through phagocytosis of foreign materials. If the foreign material persists, macrophages further differentiate and eventually fuse into foreign-body giant cells (FBGCs) comprising up to 100 nuclei [47]. These cells can create a fibrotic capsule around biomaterials, isolating them from the surrounding tissue [72]. The polarization status of macrophages is not constant and can be regulated by environmental cues [73, 74], which is called macrophage repolarization [75, 76]. Due to their remarkable plasticity and functionality, macrophages have become a hotspot in the study of immunomodulation.

### DC maturation and T lymphocyte activation by biomaterials

In addition to the innate immune system, the adaptive immune system is also involved in host response to biomaterials. DCs are professional antigen presentation cells that bridge the innate and adaptive immune systems (Fig. 2). Biomaterials based on non-natural components, such as polymers, ceramics and metals, could potentially compromise the maturation of DCs, which exhibit a tolerogenic phenotype and induce T-cell tolerance [77]. In contrast, biomaterials based on natural building-blocks, such as collagen, chitosan, agarose, hyaluronic acid (HA) and alginate can promote DCs maturation and activation [78–80]. These matured DCs promote T lymphocyte proliferation and secretion of inflammatory cytokines (TNFα and IL6), which amplify DC maturation [81]. Based on this DCs maturation, biomaterials, particularly hydrogels, are used as adjuvant for vaccine design [82, 83]. Interestingly, T lymphocytes respond to not only DCs but also macrophages, to whom they exhibit an intimate relation in biomaterial-triggered immune events. In fact, T lymphocytes can attach primarily to macrophages and promote adhesion and fusion of macrophages [84] as well as regulate macrophage polarization. The macrophage phenotype switch from M1 to M2 can be achieved through cytokine profile changes from Th1 to Th2 cell type, indicating that T lymphocytes are also potent targets for inflammation resolution and tissue regeneration [85]. In addition, T lymphocytes and macrophages can together release inflammatory mediators, which recruit inflammatory effector cells. These inflammatory factors, then decrease over time and are replaced by factors for ECM remodeling in wound healing [86–88]. These studies provide solid evidence for the ability of T lymphocyte–macrophage interactions to direct the inflammatory phase of FBR.

In summary, biomaterial design should take into account the activation of both innate and adaptive immune cells and further intricate cross-talk between cellular and soluble immune components. Consequently, the potential biocompatibility and tuneability of physicochemical properties make hydrogels promising tools for immune-instructive programming.

## Immune-instructive hydrogels by modulating physical and chemical properties

**Figure 3.**
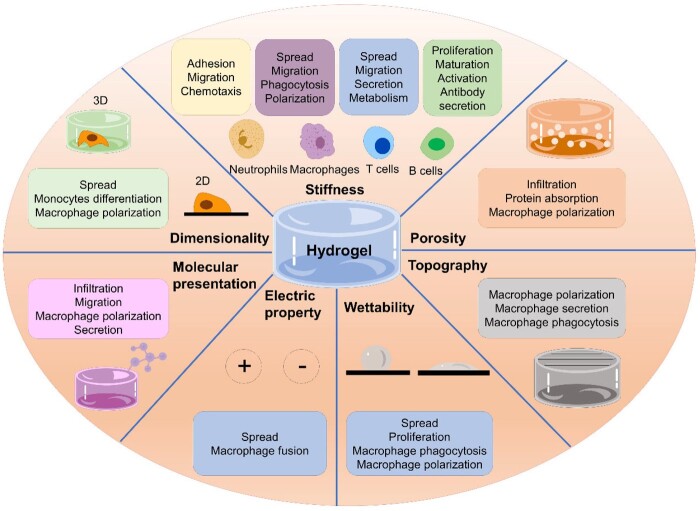
Summary of the key physical and chemical properties of hydrogels that can be used to design and control immunomodulation

Hydrogels offer the possibility to tailor their physicochemical properties. In this section, we discuss how desired immunomodulation can be designed through regulation of physical and chemical properties of hydrogels. Here, we define ‘physical properties’ as structural and mechanical properties including dimensionality (2D or 3D), stiffness, porosity and topography; while ‘chemical properties’ is defined as chemical composition including wettability, electrical properties and molecular presentation (Fig. 3).

### Physical properties

Clues to modulate biological responses through physical properties are present in nature [28]. For instance, the shape of bacteria, such as spheres, rods or spirals, influence their infection ability and recognition by mammalian immune system [89] while the stiffness of the ECM plays a key role in cancer progression, with e.g. high stiffness predicting poor prognosis in women with invasive breast cancer [90, 91]. These and many other examples suggest that physical environmental features can affect and even regulate immune responses. In this sub-section, we discuss how the physical properties of hydrogels can modulate immune responses.

#### Dimensionality

##### Dimensionality affects cell morphology

**Figure 4.**
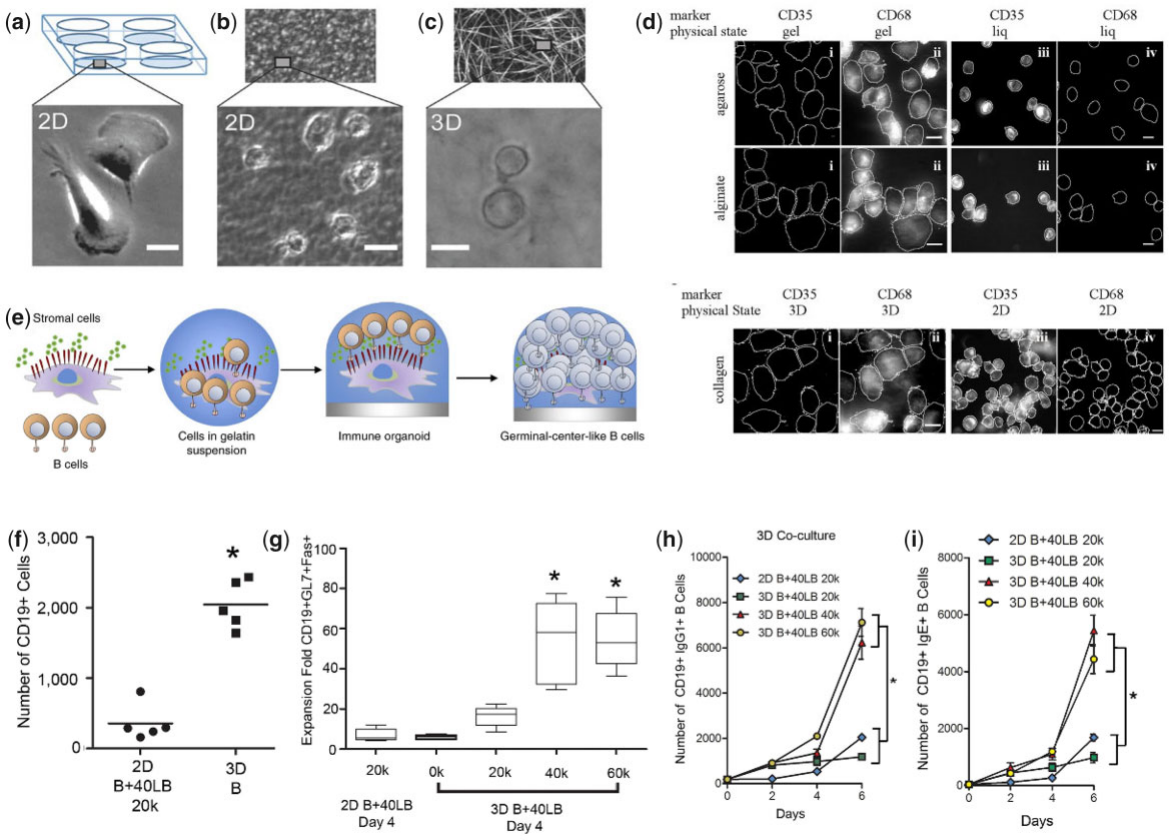
Dimensionality of hydrogels regulates immune cell behaviour. (**a–c**) Morphology of monocytes on TCPs (a), dense 2D collagen matrices (b), and 3D collagen networks (c). Adapted with permission from Ref. [93]. (**d**) Immunofluorescence images of THP-1 cells for CD68 and CD35 cultured in different micro-environment (gel like or liquid like cover of agarose and alginate, and 2D or 3D collagen matrix). Adapted with permission from Ref. [94]. (**e**) Schematic of immune organoid development with GC-like B-cell differentiation processes occurring within the 3D immune organoids over time. Adapted with permission from Ref. [99]. (**f**) Proliferation of primary B cells within the immune organoid. (**g**) *Ex vivo* induction into GC B-cell type within the immune organoid. (**h–i**) B cells cultured in 3D undergo antibody class switching to IgG1 (h) and IgE (i) more effectively than 2D. Adapted with permission from Ref. [98].

In natural tissues, cells reside in a complex 3D milieu provided by the ECM and adjacent cells. Hydrogels can be regarded as a simple version of a tissue microenvironment and the simple modification of growing on or within them can have drastic effects. Similar to other cells, immune cells cultured on and within 3D hydrogels exhibit distinct responses. For example, neutrophils growing within matrigel displayed multiple leading edge F-actin rich pseudopods, which was different from cells on 2D plastic surface exhibiting a single broad leading edge containing F-actin and a uropod [92]. Also, monocyte morphology was drastically different when grown on 2D non-coated tissue culture plastic (TCP) exhibiting spread shapes of ∼40–50 µm in diameter within 3D collagen matrices they displayed rounded shapes with diameters of ∼20–30 µm (Fig. 4a–c) [93].

##### Dimensionality affects monocyte differentiation and macrophage polarization

In addition to morphology, differentiation of monocytes and further macrophage polarization are also influenced by dimensionality. Without additional stimulus, human monocyte cells THP-1 growing in 3D gel-like environments, but not on 2D glass, could differentiate into macrophages with positively expressed CD68 (marker of macrophage) as well as increased NO and ROS production (Fig. 4d) [94]. This spontaneous differentiation was independent of gel composition and induced by a positive feedback loop associated to adhesion, which is mediated through NF-κβ pathway activation. As for macrophages growing within 3D hydrogels, the response of macrophages could be indefinite. All the M1 markers (iNOS, COX2 and TNFα) of macrophage within 3D poly(ethylene glycol) (PEG) hydrogel were up-regulated compared to those of cells growing on 2D [95]. While macrophages in 3D lymph node extracellular matrix (LNEM) hydrogel were more effectively induced into M2 than those on 2D LNEM coating, with elongated morphology and enhanced M2 marker expression and anti-inflammatory cytokines production [96]. These inconsistent results from different research could be attributed to the variation of hydrogel composition. Addition of extra stimulation results in a prominently different cell response in 3D environment. Friedemann *et al*. [93] prepared 3D collagen network to investigate the macrophage phenotype change exposed to an inflammatory microenvironment established by GM-CSF and lipopolysaccharide (LPS). The cytokine profile revealed that markers of both M1 (IL-12 and TNFα) and M2 (IL-10) were higher in cells growing on 2D matrices compared to those growing in 3D. When the ratio of IL-10 to IL-12 was used as criterion of M2 polarization, however, cells in the 3D network displayed a higher polarization tendency towards a M2 phenotype.

##### Dimensionality for immunomodulation in biomedical applications

Given the 3D tissue-like environment that hydrogels offer, they have been used with organoids to reprogram the function of existing immune tissues and for immunotherapies [97]. Recently, germinal centre (GC)-like structures were created using 3D hydrogels encapsulating B cells and fibroblasts (Fig. 4e) [98, 99]. The 3D structures (but not the 2D setup) promoted B-cells proliferation and differentiation to GC phenotype (B220^+^ Fas^+^ GL7^+^) and induced antibody class switching from IgM to IgG1 and IgE (Fig. 4f–i). Although secretion of antibodies can be induced, these GC-like structures did not produce antigen-specific antibodies. To solve this problem, Nichols *et al*. [100] developed an artificial bone marrow (BM) environment utilizing a hydrogel inverted colloidal crystal (ICC) scaffold seeded with haematopoietic stem cells (HSCs) and BM stromal cells. In this case, the 3D scaffold elevated HSC proliferation and differentiation to B cells compared to the 2D condition. When exposed to a heat-killed virus, these cells matured and released antigen-specific antibodies. These studies indicate that the three-dimensionality provided by the hydrogels is important to recreate key natural processes of immune cell development.

Overall, the 3D environment enabled by hydrogels provides cells with a biomimetic environment analogous to those of natural tissues, which have influence on cell morphology, monocyte differentiation and macrophage polarization.

#### Stiffness

##### Stiffness in tissue development and regeneration

Stiffness is defined as the ability of the material to resist deformation in response to an applied force [101]. It has become increasingly evident that most of cellular events of an organism, from early embryogenesis to tissue repair, are all influenced or regulated to some extent by tissue stiffness [102]. Therefore, tissue engineering or regenerative strategies must consider biomaterial stiffness as a key design parameter to match that of the local biological tissue and properly signal cells. Although hydrogels are generally considered as soft materials, their stiffness can be enhanced through different mechanisms, such as increased concentrations or via the use of cross-linkers.

##### Stiffness affects neutrophils

**Figure 5.**
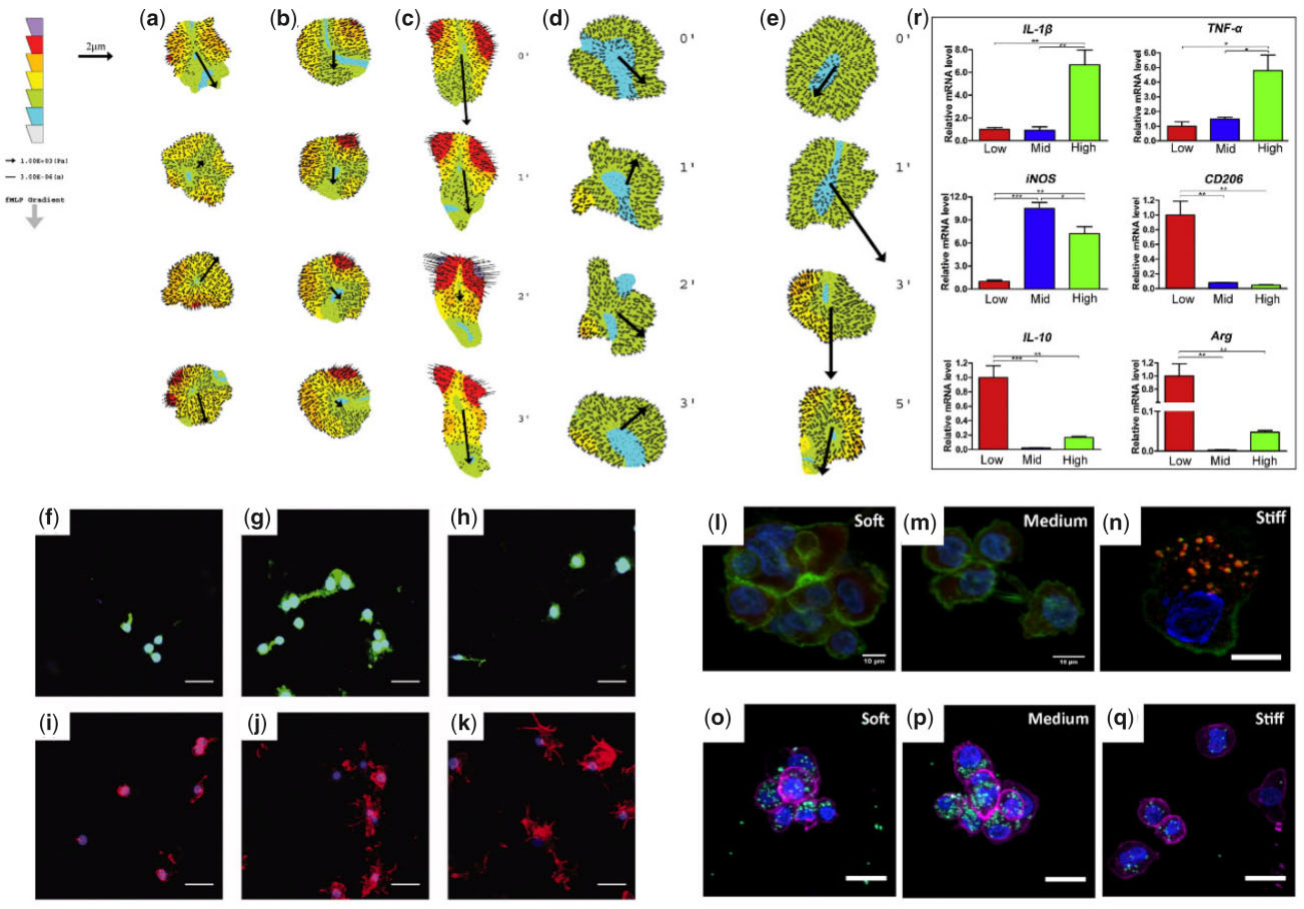
Stiffness of hydrogels regulates immune cell response. (**a–c**) Neutrophil traction stress maps on stiff hydrogels (12 kPa) in response to a uniform solution of chemoattractant fMLP (a), a shallow gradient of fMLP (b), and a steep gradient of fMLP (c). (**d–e**) Neutrophil traction stress maps on soft hydrogels (2 kPa) in response to a uniform solution of fMLP (d) and a steep gradient of fMLP (e). Adapted with permission from Ref. [105]. (**f–k)** Spatial localization of α_v_ integrins (f–h) and F-actin (i–k) in macrophages cultured on 130 (f, i), 240 (g, j), and 840 (h, k) kPa PEG-RGD gels for 48 h. Adapted with permission from Ref. [106]. (**l–n**) Confocal microscopy images of macrophages seeded in soft (l), medium (m), and stiff (n) gels. Actin cytoskeleton is represented in green, vinculin in red, and cell nuclei in blue. (**o–q**) Confocal microscopy images of macrophage phagocytosis of 1 mm latex beads in soft (o), medium (p), and stiff (q) gels. (**r**) Expression of polarization markers (Il-1b, tnf-a, and iNOS were used as the M1-polarized markers, whereas arg, CD 206, and Il-10 were used as the M2-polarized markers) of macrophages encapsulated in gels with different stiffness levels. Adapted with permission from Ref. [107].

At the onset of the FBR, neutrophils migrate to reach the inflammation site, a process within which they respond to the stiffness of the surrounding environment. Investigating this process, polyacrylamide hydrogels with different stiffness prepared via varying percentage of acrylamide and bis-acrylamide were used to study the adhesion and migration of neutrophils [103]. In this case, cell spreading was directly related to gel stiffness, with stiffer gels inducing larger spreading area. During migration, cells on soft gels (5 kPa) displayed higher speeds but moving in random directions, while on rigid gels (100 kPa) cells migrated slower but with higher directional persistence. It should be noted that the directional migration of neutrophils is trigged by response to chemokines *in vivo* [104]. Jannat *et al*. [105] investigated the response of neutrophils on hydrogels with different stiffness under well-defined chemotactic gradients generated by a microfluidic device emulating the *in vivo* scenario (Fig. 5a–e). The results demonstrated that neutrophils growing on stiff (12 kPa) hydrogels were better suited to perceive chemoattractant gradients, which resulted in asymmetrical distribution of traction forces and further induction of neutrophil polarity compared to soft (2 kPa) hydrogels. The team found that soft hydrogels weakened the neutrophil response to the chemoattractants and proposed that the absence of perception related to β2 integrin signaling.

##### Stiffness affects macrophages

The stiffness of hydrogels also affects the behaviour of macrophages. Cells on soft hydrogels maintained round shapes and displayed rapid migratory behaviours in a ROCK-dependent, podosome-independent manner. In contrast, cells on stiff hydrogels exhibited more spread morphologies with protruding filopodia and slower ROCK-independent but podosome-dependent migratory manner (Fig. 5f–n) [106, 107]. Another way in which stiffness affects macrophages is in their capacity to phagocytose. High levels of phagocytosis were observed in cells growing on soft hydrogels, while those on stiff ones exhibited a significant decrease in phagocytosis (Fig. 5o–q) [107]. This effect is also important because phagocytosis of macrophages is closely related to their polarization status. Numerous groups have studied the relation between macrophage polarization and hydrogels stiffness by controlling concentration or taking advantage of cross-linker, though the stiffnesses tested varied widely among the studies. In general, soft hydrogels have been found to induce M2 polarization, with enhanced secretion of IL-10, CD206 and Arg-1; while cells growing on stiff hydrogels were inclined to M1 polarization, expressing high levels of IL-1β, TNFα, CXCL11 and CCL20 (Fig. 5r) [107–110]. This regulatory effect of stiffness on macrophage polarization can be retained in inflammation microenvironment. As a potent agonist for M1 polarization, LPS induced increased expression of M1 markers in macrophages growing on both soft and stiff hydrogels. However, cells on soft hydrogels revealed a much lower level of increase than those on stiff hydrogels [106, 107]. More importantly, M2 markers still enhanced in cells growing on soft hydrogels under this inflammatory condition, suggesting the preference for M2 polarization. Identical with these investigations *in vitro*, soft hydrogels exhibited weak FBR in a subcutaneous implantation model, displaying an encapsulated thin layer of macrophages and a dense thin fibrous collagen capsule. In contrast, stiff hydrogels were surrounded by a large dense activated macrophage layer, which was encompassed by loose and disordered fibrous networks, indicating a stronger FBR [106].

##### Stiffness affects adaptive immune cells

In addition to innate immune cells, adaptive immune cells also respond to stiffness. Polyacrylamide gels with different stiffnesses were used to study activation of T lymphocytes [111, 112]. Compared with soft gels, stiff gels remarkably enhanced cell spreading and faster migrations with higher mean instantaneous velocities and longer maximum distances travelled. Furthermore, the gene expression and metabolism of T lymphocytes can be regulated by stiffness too. Cells on stiff gels expressed pronouncedly higher levels of IL-12, IL-22 and IFN-γ, and exhibited increased glycolytic switch, which consist with the fact that T lymphocytes exhibit a metabolic status of aerobic glycolysis upon activation [113]. Further studies revealed that this stiffness-mediated activation of lymphocytes is strongly associated with CD3 signalling [111, 112]. The activation of B lymphocytes is also regulated by stiffness. For example, Wan *et al*. [114] reported that B lymphocytes encountering an antigen-coated polyacrylamide gel with high stiffness exhibited much stronger activation. On the stiffer gel, more accumulated B-cell receptors (BCRs), phospho-spleen tyrosine kinase and phospho-tyrosine molecules were observed in the immunological synapse, as well as higher expression of the B-cell activation marker CD69 in the B lymphocytes. This mechanosensing ability of B lymphocytes is mostly dependent on their microtubule network, as proved by compromised B lymphocyte activation through treatment of a microtubule polymerization inhibitor.

##### Stiffness for immunomodulation in biomedical applications

Taking advantage of the possibility to synthesize gelatin hydrogels with different stiffnesses, Purwada *et al*. [98] investigated the effect of organoid stiffness on B-cell maturation. In order to mimic the stiffness of the GC (∼2.3 ± 1 kPa) where B cells proliferate and mature, the hydrogels were reinforced with silicate nanoparticles to exhibit a stiffness of 1.9 ± 0.5 kPa [115]. This stiffness-mimicking hydrogel provided a favourable environment for B-cell survival, differentiation and antibody secretion. In addition, stiffness also plays a key role in tissue engineering and tissue repair, where multiple cell types can respond differently to different stiffnesses. For example, it is now well known that stiff substrates induce osteogenesis of mesenchymal stem cells (MSCs), as well as M1 polarization of macrophages that will compromise the osteogenic differentiation [31]. To achieve optimal bone regeneration, He *et al.* [108, 116] prepared gelatin hydrogels with different stiffnesses and found that hydrogels of 60 kPa exhibited higher bone regeneration through synergistic effects arising from osteogenic differentiation and M2 polarization.

Overall, these studies demonstrate that the stiffness of hydrogels could affect multiple immune cells including neutrophils, macrophages and T and B lymphocytes. In general, softer hydrogels for the most part induce an immune tolerant or wound-healing phenotypes, while stiffer hydrogels promote immune-activated phenotypes.

#### Porosity

##### Porosity affects immune cell infiltration

In natural tissues, cells tend to reside within a porous ECM, which provides geometrically confined spaces and facilitates capturing of nutrients, diffusion of waste products and cell–cell communication [117]. Hydrogels with porous structures have been used to regulate immune cell responses, by enabling cell infiltration deeply inside hydrogels with larger pores [118]. However, higher porosities do not necessarily lead to higher immune cell infiltration. For example, degradable micro-scale porogen was incorporated into hydrogels to increase porosity, which lead to hydrogels with a 50% porogen fraction allowing more DCs infiltration than that with 75% and 25% porogen fractions [119]. Interestingly, these infiltrated DCs maintained an immature phenotype, which opens the opportunity to be further programmed by well-defined local cues. In contrast to microporous hydrogels, nanoporous ones could protect content from immune responses by preventing immune cell infiltration. Pro-inflammatory T lymphocytes were reported to block osteogenesis of MSCs through inducing apoptosis and down-regulating osteogenic factors [120]. Furthermore, Moshaverinia *et al*. [121] developed an MSC-laden alginate hydrogel exhibiting an average pore size of 600 nm by increasing cross-linker concentration. The nanoporous hydrogel restrained infiltration of pro-inflammatory T lymphocytes and their secreted cytokines, which reduced MSC apoptosis and promoted bone regeneration *in vivo*.

##### Porosity affects macrophage polarization

In addition to playing a role in cell migration and infiltration, porosity can also affect cell phenotype. Hydrogels with different pore sizes were implanted subcutaneously into mice to study the FBR [122]. Porous hydrogels (34-µm pore size) revealed a thinner fibrotic capsule compared to non-porous one, which suggests a mild FBR. However, macrophages infiltrated in the porous implant exhibited enhanced M1 and reduced M2 marker expression, which usually appear in severe FBR. This seemingly paradoxical phenomenon merits further investigation. Another study from the same group fabricated porous hydrogels by taking advantage of polycarbonate core/poly (methyl methacrylate) shell template that can be solubilized in dichloromethane. The hydrogels in a myocardial implantation model caused a thinner collagen capsule compared to non-porous hydrogels, with positive correlation between pore size and capsule thickness [123]. Macrophages infiltrated within the porous hydrogels expressed higher M2 marker, with 40-µm pore size hydrogels displaying the strongest effects and contributing to angiogenesis. Hydrogels with varied pore size can also be obtained through modulation of temperature and concentration. These hydrogels both showed that larger pore size had greater potential to guide M1 to M2 transition and anti-inflammatory cytokines secretion compared with smaller ones [124, 125]. The better anti-inflammatory effect of hydrogels could raise preferable angiogenic response *in vitro* and blood vessel formation *in vivo*. Desired immunomodulation effects can be achieved by simulating the porosity of natural tissues. For example, Li *et al*. [126] fabricated hydrogels with porous structures similar to a native adipose ECM. Using a rabbit inguinal fat pads defect model, the porous hydrogel induced macrophage infiltration and enhanced M2 polarization as well as angiogenesis.

Overall, hydrogel porosity has been shown to have an immunomodulatory effect through control of infiltration of immune components, such as DCs, T lymphocytes and cytokines. In addition, porosity also has effects on macrophage polarization, but results from different studies are inconclusive in terms of providing a clear effect pattern. Therefore, more research is needed to systematically investigate this evident effect.

#### Topography

##### Topography affects immune response

**Figure 6.**
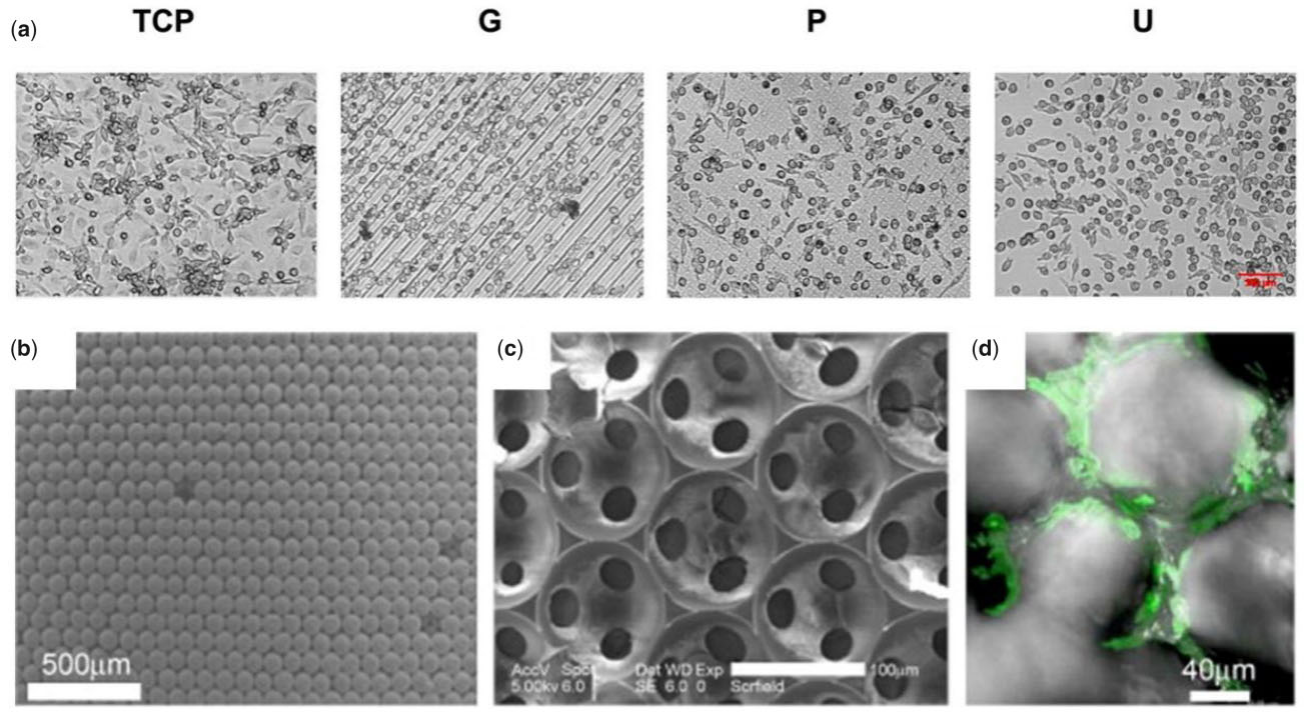
Topography of hydrogels regulates immune cell response. (**a**) Representative phase contrast images of human monocyte-derived macrophages cultured on TCP controls and GelMA micropatterns: G, microgrooves/ridges; P, micropillars; U, unpatterned for 3 days. Scale bar, 100 µm. Adapted with permission from Ref. [43]. (**b–d**) Cellular scaffolds with ICC topography. Scanning electron microscopy image of colloidal crystals made from 110-µm polystyrene beads (b), and ICC scaffolds from silicate gel (c). (d) ICC scaffolds after 5 days in culture with BM stromal cells. Adapted with permission from Ref. [100].

Another strategy to modulate immune responses is through hydrogel surface topography. Micropatterned hydrogels prepared by soft photolithography have been used to investigate macrophage responses [43] e.g. elucidating that immune cells align along microgrooved-containing hydrogels compared to other patterns or unpatterned hydrogels (Fig. 6a). Higher secretion of the M2 marker IL-1RA and phagocytic activity were also observed on cells growing on these microgrooved hydrogels compared to cells growing on hydrogels comprising micropillars. Besides micropatterns, nanopatterns also can modulate immune responses. For example, Takahashi *et al*. [127] constructed nano-patterned hydrogels by utilizing silicon moulds. Implanting the hydrogels in the back of rats triggered less fibrotic capsule compared to hydrogels with smooth surfaces. In addition, significantly less macrophage infiltration was observed into the nano-patterned hydrogels compared to the flat ones, which was attributed to a decreased cell adhesion on nano-patterned hydrogels.

##### Topography for immunomodulation in biomedical applications

The possibility to trigger desirable immune responses solely through surface topographies is a highly attractive alternative as it avoids disturbing the delicate biochemical environment. Colloidal crystals that can be removed in tetrahydrofuran were used as template to prepare hydrogels. With open geometry, full interconnectivity, high porosity and large surface area, the hydrogels exhibited topographies that resemble those of BM tissue. The BM-mimicking hydrogels promoted B lymphocyte differentiation and maturation from HSCs, exhibiting the capacity to generate antigen-specific antibodies as a result of exposure to a virus (Fig. 6b–d) [100]. Another example is the use of hydrogels as vaccines for antitumor immune responses [128]. In this effort, Xing *et al*. developed a fibrous hydrogel comprising nanofibers having a helical structure similar to fimbrial antigens that can elicit evident immune responses. Interestingly, the hydrogel was able to induce strong T-cell responses to inhibit tumour growth without antigens, immune regulatory factors or adjuvants. Immunogenicity of the hydrogel, however, was negligible, demonstrated by an insignificant change in weight and organ index among different groups. These results suggest a capacity to use parameters associated to hydrogel structure as promising tools to manipulate immune responses on demand.

Overall, while the immunomodulatory effects of hydrogel structural properties are evident, there have been limited numbers of studies providing systematic investigations, which open the possibility for discovery and improved design of hydrogel materials. Nonetheless, hydrogels are soft materials, which make it difficult to implement precise geometrical patterns on and within these materials. Therefore, new technologies are needed to facilitate incorporation of such structural features within hydrogels.

### Chemical properties

Cells in organisms are subjected to not only physical stimuli but also inductive chemical signals, which affect a diverse set of cellular events, such as adhesion, migration, proliferation and differentiation. The natural ECM provides cells with chemical simulating factors that can regulate these cellular behaviours. For example, it is known that fibronectin and laminin promote cell adhesion through the amino acid sequence arginine–glycine–aspartic acid (RGD) or isoleucine–lysine–valine–alanine–valine, respectively [129]. Moreover, growth factors and soluble signals can be sequestered and activated by glycosaminoglycans (GAGs) and proteoglycans from ECM to affect cell behaviours [130]. These factors can also be triggered e.g. via electrical stimulation that can cause protuberances on the presynaptic membrane of nerve terminals, leading to the release of neurotransmitters [131]. Therefore, control of chemical signalling represents a powerful opportunity for hydrogels to tailor the immune microenvironment. In this sub-section, we highlight the significance of chemical properties of hydrogels in immunomodulation.

#### Wettability

Depending on the chosen building-blocks, hydrogels can be hydrophilic or hydrophobic. It is widely recognized that surface wettability regulates cell adhesion [132]. Macrophages on hydrophobic polystyrene surface revealed an M2-like phenotype but exhibited M1-like phenotype on hydrophilic O_2_ plasma-etched polystyrene surfaces [133]. Analogously, macrophages have been reported to preferentially attach to hydrophobic silicone surfaces but higher levels of expression of inflammatory factors, such as TNFα and IL-1β have been observed on macrophages growing on hydrophilic PEG hydrogels [134]. However, hydrogels and silicones are two entirely different materials with many differences, which make this finding inconclusive as a result of confounding variables. Therefore, Xu *et al*. [44] fabricated methacrylated gellan gum (MGG) hydrogels with different levels of hydrophobicity by attaching hydrophobic alkyl branches to MGG without changes in hydrogel stiffness and composition. In this case, hydrophobic hydrogels promoted cell proliferation and spreading, which may be associated with increased protein absorption on hydrophobic surfaces [135]. The production of cytokines associated with M1 and M2 phenotypes were enhanced by cells growing on hydrophobic hydrogels, with M1 cytokine secretion being more pronounced, suggesting a classical phenotype shift of macrophages with increasing hydrogel hydrophobicity. In addition, the function of macrophages can also be tailored by gel wettability. For example, cells on hydrophilic surfaces have been shown to exhibit lower phagocytosis rates, evidencing the importance of hydrophobicity in effective phagocytosis [136].

#### Electric property

**Figure 7.**
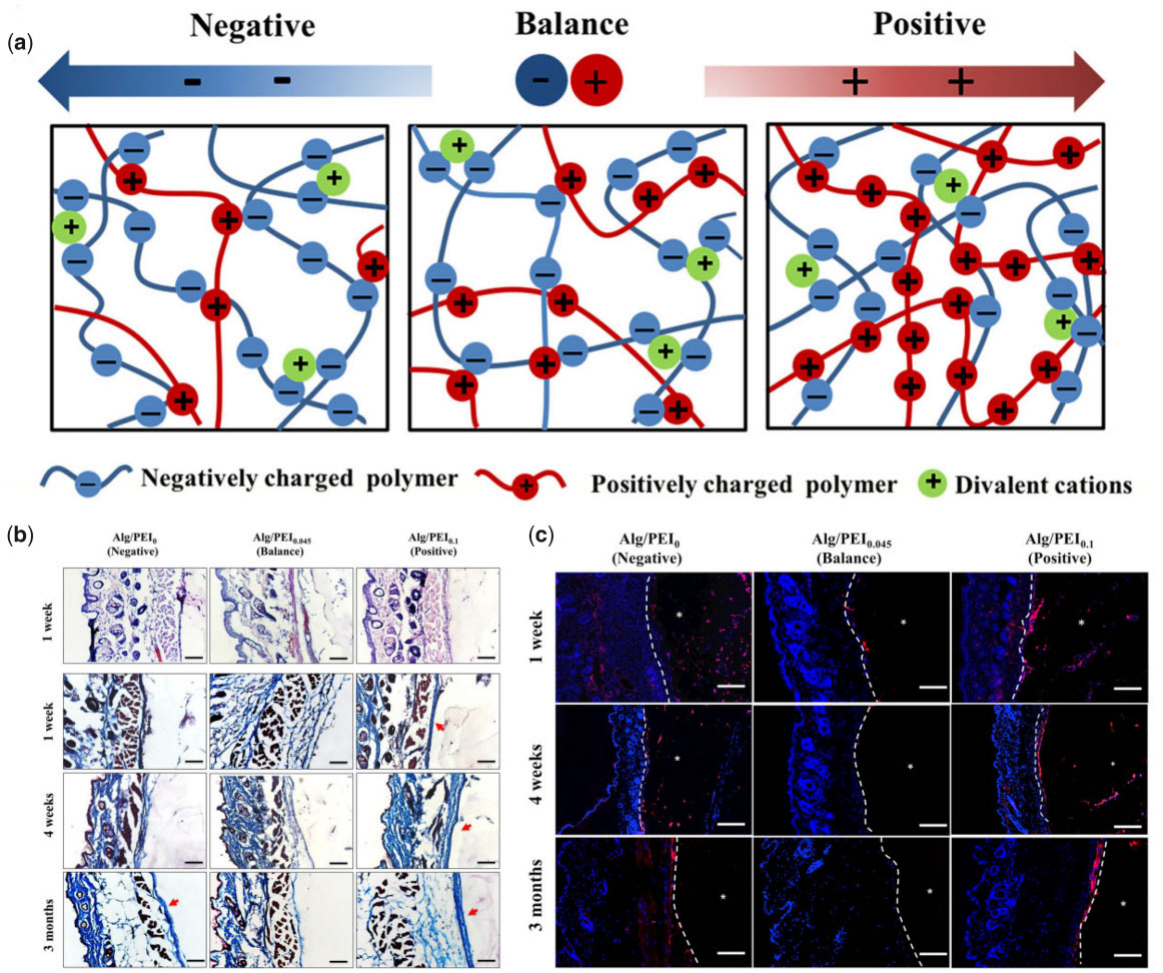
Electric property of hydrogels regulates immune cell response. (**a**) Hydrogel preparation based on oppositely charger polyelectrolytes assembly. (**b–c**) Inflammatory responses, capsule formation (b) and macrophage migration and infiltration (c) after subcutaneous implantation in mice. Adapted with permission from Ref. [144].

Electrical signals are involved in almost all functions of living cells and organisms [137, 138]. Physiological electrical fields existing in injury sites have been proposed to play an essential role in wound healing [139, 140]. Furthermore, it has been reported that the surface charge density of implants can influence amount and conformation of absorbed proteins, which further affect immune responses [141, 142]. Through the modification of functional groups, hydrogels can be designed to display different surface charges [143]. Positively charged hydrogels have been found to induce macrophage spreading, while negatively charged ones inhibited spreading. In addition, the fusion of macrophages into FBGCs was more obvious on positively charged hydrogels in comparison with negatively charged one. This enhanced cell spreading and fusion could be attributed to electrostatic interactions between the positively charged hydrogel surface and negatively charged cell membranes. Other than modification of functional groups, hydrogels with varied electric property also could be acquired by regulating ratio of alginate (negative charged) and PEI (positive charged) [144]. In a subcutaneous rat model, balanced charged (neutral) hydrogel effectively mitigated FBR and delayed capsule formation for at least 3-months, while positively charged one displayed the strongest stimulation effects (Fig. 7) [144]. Similarly, a study using multidomain peptide (MDP) hydrogels reported that positively charged MDP K_2_(SL)_6_K_2_ and R_2_(SL)_6_R_2_ peptides also elicited pronounced FBR, characterized by multiple immune cells infiltration and high degree of collagen deposition [145]. In contrast, negatively charged MDP E_2_(SL)_6_E_2_ and D_2_(SL)_6_D_2_ were infiltrated by tissue-resident macrophage at low extent and revealed no collagen deposition, suggesting a low level of FBR.

#### Molecular presentation

Hydrogels can be classified by various methods, such as cross-linking property, preparation methods, degradability, sources, and so on. In terms of sources, hydrogels can be fabricated from both natural and synthetic materials for biomedical application. Naturally-derived hydrogels, such as ECM, protein, peptides and nucleic acids, are attractive because of their cell interactive and cell signalling features, and biodegradability. However, their shortcomings include difficulties in controlling structure and degradation, low mechanical properties and potential contamination from their source. On the other hand, hydrogels from synthetic sources usually have controllable structural units, and result in controllable degradation and mechanical properties, but are short of bioactive moieties. Through controlling their chemical composition, hydrogels present various molecules or functional groups to cells, which cause different immune responses.

##### Decellularized matrices affect immune response

**Figure 8.**
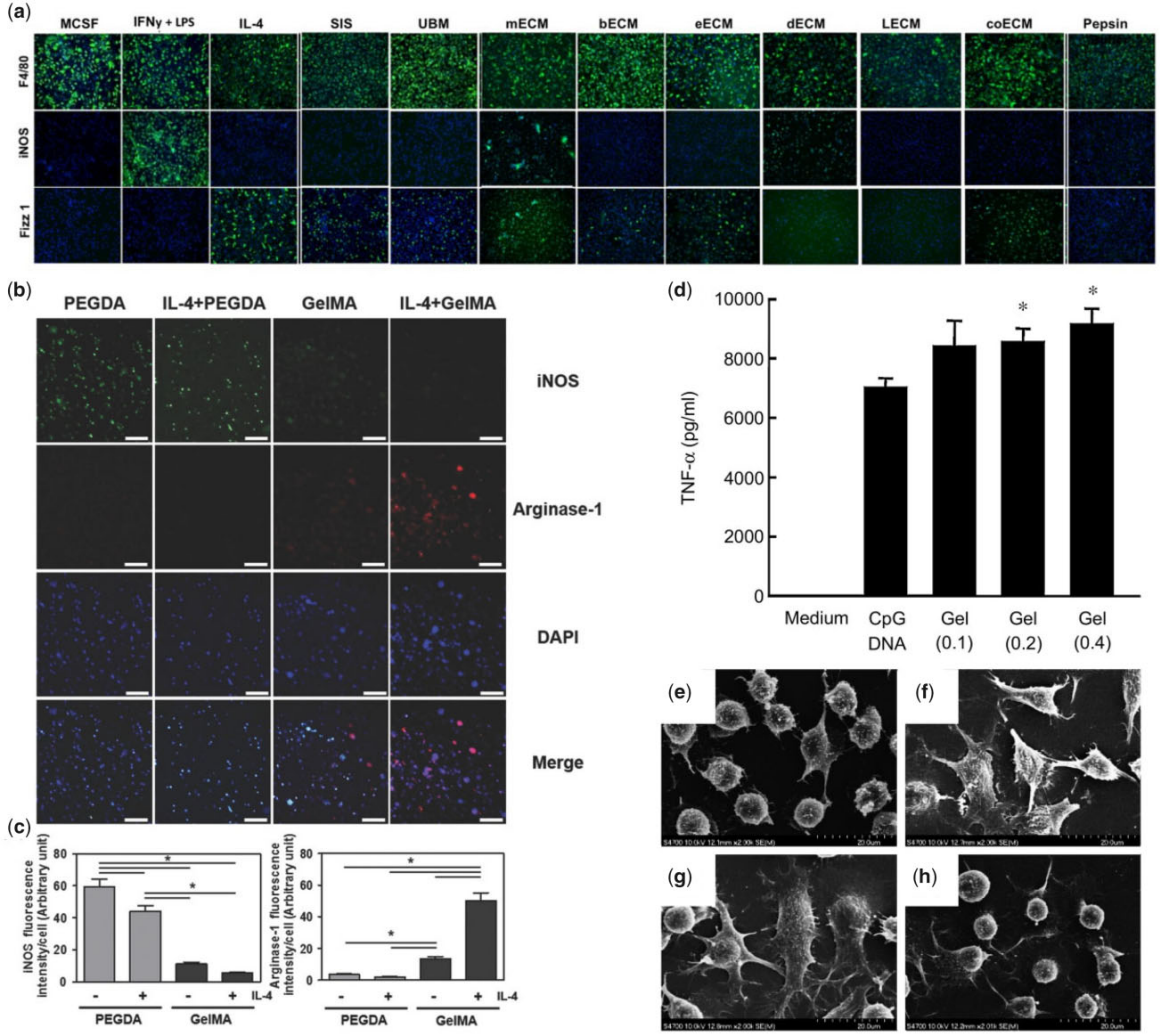
Molecular presentation of hydrogels regulates immune cell response. (**a**) Immunofluorescence of different ECM treated macrophage for M1 and M2 phenotype (iNOS and Fizz1, respectively). F4/80 was used as a pan macrophage marker. Adapted with permission from Ref. [148]. (**b**) Immunofluorescence of macrophage encapsulated in PEGDA and GelMA hydrogels in the absence or presence of IL-4 for M1 and M2 phenotype (iNOS and arginase-1, respectively). (**c**) Quantitative analysis of (b). Adapted with permission from Ref. [21]. (**d**) TNF-α production from RAW264.7 cells after addition of CpG X-DNA (CpG DNA) or CpG DNA hydrogels. (**e–h**) SEM images of DC2.4 cells before (e) and after addition of LPS (f), CpG DNA hydrogels (g), or CpG-free DNA hydrogels (h). Adapted with permission from Ref. [161].

It is widely recognized that the ECM contains natural immunomodulatory domains capable of binding to specific receptors expressed on immune cells, which promote their adhesion and regulate their function [146]. Therefore, ECM-based hydrogels can be used as effective biomaterials to direct desirable immune responses. Decellularized matrices are fabricated by removing cells from tissue through detergents or other cell lysing processes, while maintaining most ECM components. Using a decellularized small intestinal submucosa (SIS), Sicari *et al*. [147] demonstrated the capacity to promote M2 polarization of macrophages through the matrix composition rather than its structural or mechanical properties, evidenced by high levels of Fizz1 and CD206 expression and reduced iNOS expression. Importantly, another study demonstrated that the tissue source of decellularized matrices can affect immune responses. As reported by Dziki *et al*. [148], ECM from SIS, urinary bladder, brain, oesophagus, and colon induced M2-like macrophage phenotypes (iNOS^−^/Fizz1^+^/CD206^+^) while dermal ECM triggered M1-like phenotype (iNOS^+^/Fizz1^−^/CD206^−^) and liver and skeletal muscle ECM did not elicit macrophage polarization (Fig. 8a). Furthermore, hydrogels made from decellularized ECMs from porcine nerves were used to test peripheral nerve repair in a rat sciatic nerve defect model and found increased M2-like macrophage infiltration, suggesting immunomodulation effects taking place during the nerve repair process [149]. In another study on bone regeneration, periosteal ECM was used to manufacture hydrogels [46] that induced macrophage migration and expression of CD206 and Arg1, as well as reduced iNOS and IL-1β expression *in vitro*. In a calvarial defect model, the hydrogel triggered M1 to M2 transition of macrophages by Day 3 after implantation, which provided an osteogenesis friendly environment to promote bone repair. While decellularized ECM hydrogels are promising alternatives for tissue regeneration, there remains an important challenge to accurately control their molecular composition as well as potential contaminant molecules, such as residual DNA or antigenic proteins [150].

##### Molecularly designed matrixes affect immune response

In an effort to both recreate the functional complexity of the ECM and control its composition, hydrogels based on ECM components are being developed. For example, cross-linked gelatin (denatured collagen) modified with photosensitive methacryloyl groups (GelMA) was used to fabricate collagen-based hydrogels that promoted lower inflammatory factor TNFα expression by monocytes after LPS stimulation, compared with cells on TCP [151]. The hydrogel reduced soluble TNFα in the supernatant by serving as a TNFα sink, indicating a sufficient anti-inflammatory effect. Another study from the same group reported that macrophages on the same GelMA hydrogel exhibited a M2-like phenotype with or without IL-4 stimulation while macrophages on polyethylene glycol diacrylate (PEGDA) hydrogels did not (Fig. 8b and c) [21]. The immunomodulation effect of GelMA could be attributed to its abundant integrin-binding sites, which could promote macrophage adhesion and further polarization. Other hydrogels based on ECM components, such as fibrin and HA, have also exhibited immunomodulation effects [20, 152]. In particular, HA-based hydrogels have been shown to affect immune cells depending on their molecular weight (MW) with high-MW HA being inert or immunosuppressive while low-MW HA promoting inflammatory responses [153].

##### Peptides affect immune response

The ability of ECM proteins to regulate immune cell behaviour relies primarily on bioactive amino acid sequences that selectively interact with them and that can be used as building-blocks of immunomodulatory biomaterials. The use of these small bioactive sequences avoids difficulties of control of structural and conformational aspects of the ECM proteins. Therefore, a variety of hydrogels based on ECM-inspired peptides have been investigated for modulation of immune responses. The peptide RGD, identified as a minimum cellular binding motif, is one of most commonly used peptides in hydrogels [154]. A study using RGD-modified PEG (PEG-RGD) hydrogels reported that the gene expressions of Tnfα and Il1b in macrophages were less on PEG-RGD compared to PEG alone, meaning the RGD incorporation can endow hydrogels with anti-inflammatory properties [134, 155]. Subcutaneous implantation of the PEG-RGD hydrogel gave rise to a pro-inflammatory cell layer of a ∼20–40 µm in thickness, while the PEG hydrogels yielded pro-inflammatory layer thicknesses of 100–200 µm, indicating reduced FBR by exposure of the RGD peptide. In addition to natural peptides, synthetic peptides are also being used to modulate immune responses. For example, *N*-formyl-*L*-methionyl-*L*-leucyl-*L*-phenylalanine (fMLF), a tripeptide designed according to metabolic feature of bacterial, is commonly used as chemoattractant for neutrophils and macrophage [156, 157]. Hydrogels composed of fMLF and dipeptide LΔF were observed to recruit macrophages at higher levels compared to the LΔF only hydrogel [158]. To increase gelation, fMLF-derived molecules were prepared with the sequence *N*-formyl-*L*-methionyl-*L*-leucyl-*L*-3-(2-naphthyl)-alaninyl-D-3-(2-naphthyl)-alanine [159], resulting in attraction of neutrophils to desired locations *in vivo* in a sustained manner and promoting ROS production, indicating a strong potential in cancer treatment.

##### Nucleic acids affect immune response

In addition to ECM-based molecules, nucleic acids are another kind of natural components that are responded by immune cells. Nucleic acid hydrogels have been prepared by multiple methods, such as enzyme ligation, polymerization and hybridization. DNA containing unmethylated cytosine–phosphate–guanine (CpG) dinucleotides (CpG DNA) motif, which is recognized by Toll-like receptor 9 (TLR 9) expressed in innate immune cells, can be used in cancer immunotherapy [160]. CpG DNA hydrogel, constructed by DNA ligase, boosted the production of TNF-α by macrophage-like RAW 264.7 cells and maturation of dendritic DC2.4 cells (Fig. 8d–h) [161]. After incorporation with doxorubicin, the combination showed better anti-tumour capability in mice. However, this hydrogel could be contaminated by ligase, triggering unwanted response. Therefore, the ligation-free DNA hydrogels were developed through sequence-directed hybridization by same research team [162]. Immunostimulatory effect of the updated hydrogels was proved by dramatic IL-6 release from DC2.4 cells and enhanced immune response to ovalbumin integrated in hydrogel. Decreased local or systemic adverse reactions were observed in ovalbumin/hydrogel than ovalbumin injected with complete Freund’s adjuvant or alum, demonstrating the high biocompatibility. Immunoinhibitory effect of DNA hydrogels can also be achieved through modulating the sequence of nucleic acids. Nishida *et al.* [163] constructed immunoinhibitory oligodeoxynucleotides into Kanji character Takumi-like structure, then incorporated into hydrogels. Pro-inflammatory cytokines released from RAW 264.7 and DC2.4 cells after stimulation by TLR 9 ligand were restrained by the hydrogels, suggesting their potential for autoimmune diseases treatment.

##### Non-natural molecules affect immune response

Although natural molecules provide excellent biocompatibility and signalling capacity, their synthesis can be limited given their precise amino acid or nucleotide sequence. Non-natural molecules, such as synthetic polymers with accurately tailored physicochemical characteristics offer a higher level of versatility and can be designed to have immunomodulatory functionalities. In addition, with reliable material sources and long shelf lives, synthetic polymers can be manufactured on a large scale with low cost. However, their application is limited by their own intrinsically poor bioactivity and noxious by-products. For example, commonly used synthetic polymers, such as polyurethane, polyethylene, poly(methyl methacrylate), poly(2-hydroxyethyl methacrylate) (PHEMA) and PEG are known to trigger strong FBR *in vivo* [164]. To overcome this problem, one approach aims to prepare hybrid hydrogels from both natural and synthetic polymers, in which immunomodulation can be provided by the natural component. For example, biopolymer gelatin together with synthetic polymer six-arm star-shaped poly(ethylene oxide-stat-propylene oxide) prepolymer with isocyanate end groups (NCO-sP(EO-stat-PO)) was used for preparation of immunomodulatory hydrogel nanofibers [165]. The macrophages on the hydrogel showed up-regulated prohealing genes and down-regulated pro-inflammatory genes, which may resulted from RGD sequence in gelatin and modulation of fibrous topography and stiffness caused by NCO-sP(EO-stat-PO). Alternatively, bio-inspired synthetic polymers can induce a desired immune response. Carboxybetaine, which is structurally similar to glycine betaine found in humans and known to play a key role in osmotic regulation, was used to fabricate poly(carboxybetaine methacrylate) (PCBMA) hydrogels [166]. Upon subcutaneous implantation in mice, these hydrogels induced less inflammation at the tissue-hydrogel interface compared to PHEMA hydrogels. Furthermore, loosely distributed collagen, resembling more a natural ECM than a foreign-body capsule, was found surrounding the PCBMA hydrogels, while PHEMA hydrogels were encapsuled by dense collagen even after 3 months of implantation. Further studies revealed that the delicate FBR caused by PCBMA hydrogels could be ascribed to macrophages expressing anti-inflammatory factors. In addition to synthetic molecules, immunomodulatory effects of functional groups are also investigated. For example, Vegas *et al*. [167] synthesized 774 alginate analogues through modification of alginate with a variety of functional groups including amines, alcohols, azides, and alkynes. The team reported that hydrogels from triazole-containing analogues induced decreased FBR in both mice and non-human primates, with less immune cell infiltration and thinner collagen capsules for at least 6 months. These results suggest that triazole modifications may have broad implications in biomedical applications. Interestingly, gases can also be used to modulate immune responses. Fabricating a hydrogel by combining HA and JK1, a hydrogen sulphide (H_2_S) donor, Wu *et al*. [168] reported that H_2_S released from the hydrogels induced macrophage M2 polarization and promoted wound repair.

### Delivery of bioactive molecules

Hydrogels can exhibit properties, such as high biocompatibility, porosity and ease of modification, which make them desirable scaffolds to incorporate exogenous bioactive molecules. As a result, hydrogels can modulate immune responses through controlled release of immunomodulatory components by tuning their physical and chemical properties.

#### Controlled release by pore size affects immune response

Pore size of hydrogels not only controls the immune cell response directly, but also regulates the release and retention of bioactive molecules, leading to desired immunomodulation. Kim *et al.* [169] prepared chitosan-based hydrogels with different pore size, which incorporated with anti-inflammation reagent epigallocatechin gallate for skin regeneration. With larger pore size, the hydrogel showed more proportion release of epigallocatechin gallate, resulting in reduced inflammation response of macrophage and further effective skin regeneration.

#### Controlled release by electrostatic interaction affects immune response

Because of electrical characteristic of nucleic acids, electrostatic interaction is commonly used for their loading. Leach *et al.* [170] prepared a peptide hydrogel to deliver immunomodulatory cyclic dinucleotides by electrostatic interaction between negative thiophosphate linkages of cyclic dinucleotides and the positive lysine residues at the peptide termini. The electrostatic interaction provided controlled release of cyclic dinucleotides, exhibiting 8-fold slower release rate than that of collagen hydrogel, and dramatically improved survival in mice model of head and neck cancer. In another research, an injectable hydrogel was developed to load exosomes, extracellular vesicles carrying bioactive proteins and nucleic acids that are produced by cells to regulate cell response remotely [171]. Exosomes were tethered within hydrogel via electrostatic interaction between positively charged hydrogel and negatively charged exosomes membrane, prolonging the time in which exosomes release and further exert effects. The extended release of immunomodulatory exosomes brought about M2 polarization-assisted angiogenesis and diabetic wound healing.

#### Controlled release by affinity between heparin and chemokines affects immune response

Chemokines bind GAGs from the ECM, such as heparin. By taking advantage of this feature, heparin-based hydrogels were used to deliver chemokines to modulate the local immune microenvironment. For example, using a heparin and PEG diacrylate hydrogel, Krieger *et al*. [172] enabled loading and sustained release of SDF-1α, taking advantage of its capacity to bind heparin, leading to the recruitment of innate immune cells involved in microvascular network growth. On the other hand, the affinity of heparin to chemokines can be used to scavenge excessive chemokines in inflammatory environments. For instance, a heparin-incorporated hydrogel was used to capture the inflammatory chemokines MCP-1, IL-8, MIP-1α and MIP-1β from wound fluid of chronic venous leg ulcers in patients and blocked the migratory ability of monocytes and neutrophils [173]. Furthermore, in mice delayed wound-healing model, the hydrogel performed as a scavenger of inflammation, generating an immuno-friendly microenvironment for wound closure.

#### Controlled release by environmental responsive degradation affects immune response

**Figure 9.**
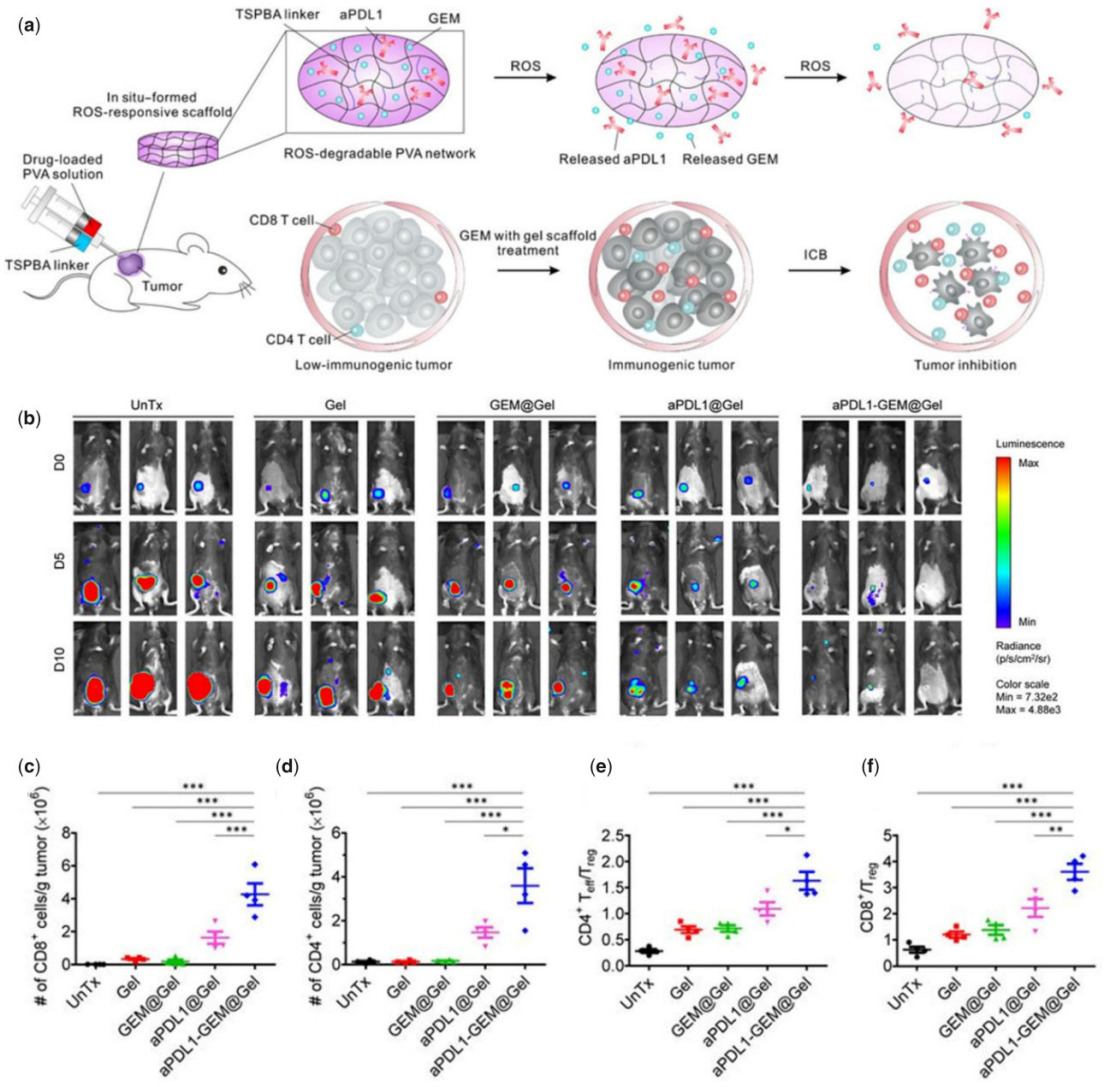
Delivery of aPDL-1 by ROS-responsive hydrogel for immunotherapy of tumour. (**a**) Schematic of combination chemoimmunotherapy using a ROS-degradable hydrogel scaffold to deliver GEM and aPDL1 into the tumour microenvironment. (**b**) *In vivo* bioluminescence imaging of the B16F10 tumour in control and treated groups. (**c–d**) Absolute numbers of the CD8^+^ (c) and CD4^+^ T cells (d) per gram of the tumour upon various treatments. (**e–f**) Ratios of the tumour-infiltrating CD8^+^ T cells (e) and CD4^+^ T cells (f) to Tregs in the tumours upon various treatments. Adapted with permission from Ref. [176].

The ECM is not only highly complex in composition but also highly dynamic. Hydrogels exhibit signals with temporal control, including the possibility to use environmental cues to tailor degradability and, consequently, the immune response. Triglycerol monostearate, an amphiphile with an ester linkage that is cleavable by esterases and matrix metalloproteinases in inflammatory conditions, was self-assembled into hydrogels to encapsulate immunosuppressive tacrolimus [174]. The release of tacrolimus from the hydrogel was observed only in the presence of inflammation-like conditions. Using a Brown Norway-to-Lewis rat hindlimb transplantation model, local injection of the hydrogel resulted in reduced immune cell infiltration and anti-graft antibody production, leading to prolonged graft survival. The distinction of pH among different tissues can also be used for controlled release. Li *et al.* [175] designed an injectable hydrogel encapsulating mesoporous silica nanoparticles-loaded miR-21-5p (MSN/miR-21-5p), with MSN/miR-21-5p conjugating within hydrogel through pH-responsive Schiff base bonds. In the slightly acidic environment of myocardial infarction, the cleavage of Schiff base bonds elicited release of immunomodulatory MSN and angiogenic miR-21-5p, inhibiting inflammatory response and promoting local neovascularization. As for cancer treatment research, a ROS-responsive hydrogel was fabricated with poly(vinyl alcohol) and a ROS labile linker (Fig. 9a) [176]. In the tumour site, abundant ROS induced degradation of the hydrogel that locally released gemcitabine (GEM) and anti-PD-L1 blocking antibodies (aPDL-1), triggering marked infiltration of CD4^+^ and CD8^+^ T lymphocytes and even systemic immune responses that inhibited tumour growth and recurrence (Fig. 9b–f).

## Future perspectives

Hydrogels continue to be promising biomaterials for a wide spectrum of applications, generating increasing interest due to their high biocompatibility, tuneability, easy of modification and controllable properties [177]. In this review, we have summarized strategies to modulate immune responses through the manipulation of physical and chemical characteristics of hydrogels (Table 1). These tactics are being employed in tissue engineering, cancer treatment, immune rejections remission, cell therapies, medical device design, and immune-related diseases amelioration. We have attempted to dissect and identify key hydrogel features that could be used as design tools for the engineering of advanced immunomodulatory hydrogels. Furthermore, emerging technologies, such as bioprinting, high-throughput screening, organoids, and organ-on-chips, are likely to enhance the repertoire of tools to tailor immunomodulation. Nonetheless, important challenges need to be considered and addressed to implement immunomodulation as a widespread feature in the design of the next generation of hydrogels:

**Table 1.** Summary of the hydrogel-based regulation strategies on immunomodulation

Property	Hydrogel example	Cell function	References
Dimensionality	Agarose gel	3D induced monocytes differentiation	[94]
	Collagen gel	3D promoted M2 polarization in inflammatory condition	[93]
Stiffness	Transglutaminase cross-linked gelatin gel (1.58 and 60.54 kPa)	Macrophages polarization Stiffer-induced M1 markers Softer-induced M2 markers	[108]
	PEG-RGD hydrogel (130, 240 and 840 kPa)	Inflammatory cytokines secretion by macrophages Stiffer promoted higher pro-inflammatory cytokines	[106]
	Polyacrylamide gel (11, 88 and 323 kPa)	Phagocytosis and migration of macrophages Softer induced highly phagocytic and migratory phenotype	[107]
	Polyacrylamide gel (10, 25, 100 and 200 kPa)	T lymphocytes activation Stiffer induced fast migration, pro-inflammatory factor expression, and aerobic glycolysis	[111–113]
	Polyacrylamide gel (2.6, 7.4 and 22.1 kPa)	B lymphocytes activation Stiffer promoted accumulated BCR and high expressed CD69	[114]
Porosity	Alginate hydrogel	50% porosity allowed more DCs infiltration	[119]
	Poly(2-hydroxyethyl methacrylate-co-methacrylic acid) hydrogel	40-µm pore size induced M2 polarization	[123]
Topography	Polyacrylamide hydrogel	Nano-pattern induced low level of FBR	[127]
	Gelatin methacryloyl hydrogel	Microgrooved pattern promoted M2 marker expression and phagocytosis	[43]
Wettability	Methacrylated gellan gum hydrogel	Hydrophobicity induced high M1 marker expression and low phagocytosis	[44]
Electric property	Alginate/PEI hydrogel/multidomain peptide (MDP) hydrogel	Positive charge elicited high level of FBR	[144, 145]
Molecular presentation	Decellularized matrices	Macrophages polarization depended on tissue source of decellularized matrices	[146]
	ECM molecules	Lower expression of pro-inflammatory factors by monocytes M2 polarization	[21, 151]
	Peptides	Recruitment of neutrophils and macrophages Decrease of inflammatory factor expression by macrophages	[134, 155, 158, 159]

### Identification of key hydrogel properties in immunomodulation

It has been widely recognized that hydrogel stiffness has a conspicuous effect on immune cell responses. In addition, stress relaxation, another mechanical cue that characterizes the ability of a substrate to dissipate cell-induced forces, has been long neglected. ECM and tissues, such as brain, liver, muscle, skin and breast are not just elastic, they are viscoelastic, exhibiting stress relaxation [178]. Viscoelastic hydrogels can provide a microenvironment that more closely simulates the natural ECM. Recently, hydrogels with different stress relaxation profiles were used to explore responses of MSCs, chondrocytes, and myoblasts [179–181]. Nevertheless, immunomodulatory effect of stress relaxation remains under explored. Therefore, identifying the relationship between stress relaxation and immune cell responses will likely lead to a new research direction contributing to building up mechanically controllable hydrogels for immunomodulation. In addition, in order to identify the effect of specific hydrogel properties on the immune response, it is critical to develop ways to control hydrogel design parameters individually. For example, to modulate the stiffness of alginate hydrogels, different concentrations of CaCl_2_ can be added [182]. However, while this approach can modulate hydrogel stiffness, it would also result in changes in porosity and composition, making it difficult to distinguish the dominant properties that influence the immune response. Consequently, it is important to develop methodologies that enable regulation of hydrogel parameters in an isolated manner.

### The mechanisms of hydrogel immunomodulation need to be investigated

Although desirable immune responses can be obtained by regulating physical and chemical properties of hydrogel, an in-depth understanding of how the changes of these properties affect immune responses is scanty. For example, effects in the cytoskeleton as a result of hydrogel properties can lead to reduced immunosuppressive effects of MSCs [183] or effects on B lymphocyte activation [114]. These effects are likely associated with distinct mechanosensing, which would depend on both type of hydrogel and cells. Furthermore, microRNAs are also implicated in hydrogel mediated immune responses [184]. However, it is necessary to elucidate these mechanisms to inform both hydrogel design and clinical application.

### High-throughput screening for combinatorial effects of hydrogel properties

The immunomodulation effects of hydrogels are not the result of single factors but rather of multiple physical and chemical properties working together. While an understanding of the effects of specific hydrogel properties would improve our understanding of fundamental mechanisms, synergistic effects are likely to define the ultimate hydrogel performance. Therefore, it is necessary to implement high-throughput screening techniques that will help to dissect and identify desirable parameters. Addressing this need, Rostam *et al*. [185] identified materials with the ability to regulate macrophage phenotype by two rounds of high-throughput screening from homopolymer and copolymer libraries. Machine-learning approaches were then used to develop polymer structure-cell response models, which could predict immune-instructive features of potentially new materials yet to be synthesized. In another study, a high-throughput combinatorial screening of biochemical and physical signals of hydrogels was used for stem cell-based cartilage tissue regeneration [186]. The study investigated integrated effects of matrix degradation, stiffness, growth factor concentration, RGD presentation, and mechanical stimulation on MSC differentiation into articular or hypertrophic cartilage phenotypes. This technique allows to conveniently and quickly screen specific properties to guide the design of immune-instructive hydrogels.

### Improved classification methods

As the understanding of immune responses to biomaterials grows, it is increasingly necessary to find ways to quantify and characterize these biological processes. As mentioned, macrophages can be typically classified into M1 (pro-inflammatory phenotype) and M2 (wound-healing phenotype) according to their function and cytokines profiles. However, increasing evidence shows that macrophages can express both M1 and M2 makers simultaneously and there is a spectrum of macrophage polarization depending on the nature of stimuli [187], making it difficult to define the activation state of these cells. For example, studies have demonstrated that macrophages can express M2 makers while at the same time produce M1 cytokine secretions depending on the hydrogel used [43, 188]. Therefore, detection protein and gene expression alone is not sufficient to determine the actual immune state. Such advances are likely to require multidisciplinary efforts integrating cell biology, immunology, materials science, and bioengineering.

### Improved understanding of the relationship between immunomodulation and immune cell metabolism

Recent advances in cell metabolism studies have reinforced the understanding of metabolic modulation affecting the immune response. For example, the transition of macrophage phenotype from M2 to M1 is concomitant with metabolic reprogramming from fatty acid oxidation to glycolysis [189]. However, studies investigating the role of hydrogel biomaterials on the metabolic state of immune cells are rare. We believe that understanding how hydrogel-derived cues, including physical and chemical properties, impact immune cell behaviour can play a key role in the ultimate immune response. This is a new outlook at immunomodulatory materials and can help us comprehend immunomodulation mechanisms and design novel immune-instructive hydrogels.

## Conclusion

The host response to biomaterials, particularly to hydrogels, implicates intricate interaction between innate and adaptive immune system. A variety of strategies have been adopted to achieve favourable hydrogels by modulating interaction at immune cells/hydrogels interface, including control of physical and chemical properties. Besides, the regulation of these inherent properties can influence delivery of bioactive molecules by hydrogels, which also affect immune response. Ongoing research is uncovering more details about the role of inherent hydrogel cues in immunomodulation. More work is needed to acquire further in-depth insight into immune cell-hydrogel biology, as well as developing new techniques to more precisely and quickly investigate the immune response to hydrogels. This knowledge will further aid the identification of design rules for the engineering of advanced immune-instructive hydrogels.

## Funding 

The work was supported by the ERC Proof-of-Concept Grant (MINGRAFT), the AO Foundation Grant (AOCMF-17-19M), the Medical Research Council (UK Regenerative Medicine Platform Acellular/Smart Materials-3D Architecture, MR/R015651/1), the National Natural Science Foundation of China (81870741, 82001023), China Postdoctoral Science Foundation (2019M661177), Natural Science Foundation of Liaoning Province (2020-MS-154) and China Scholarship Council ([2020]50).

*Conflict of interest statement*. None declared. 

## References

[1] Pollard JW. Trophic macrophages in development and disease. Nat Rev Immunol 2009;9:259–70. 19282852 10.1038/nri2528 PMC3648866

[2] Krenkel O , Tacke F. Liver macrophages in tissue homeostasis and disease. Nat Rev Immunol 2017;17:306–21. 28317925 10.1038/nri.2017.11

[3] Neves J , Zhu J, Sousa-Victor P, Konjikusic M, Riley R, Chew S, Qi Y, Jasper H, Lamba DA. Immune modulation by MANF promotes tissue repair and regenerative success in the retina. Science 2016;353:aaf3646. 27365452 10.1126/science.aaf3646 PMC5270511

[4] Gea-Banacloche JC. Immunomodulayion. In: *Principles of Molecular Medicine*. New York: Humana Press, 2006, 893–904.

[5] Sathish JG , Sethu S, Bielsky M-C, Haan L, French NS, Govindappa K, Green J, Griffiths CEM, Holgate S, Jones D, Kimber I, Moggs J, Naisbitt DJ, Pirmohamed M, Reichmann G, Sims J, Subramanyam M, Todd MD, Laan JWVD, Weaver RJ, Park BK. Challenges and approaches for the development of safer immunomodulatory biologics. Nat Rev Drug Discov 2013;12:306–24. 23535934 10.1038/nrd3974 PMC7097261

[6] Singh A , Peppas NA. Hydrogels and scaffolds for immunomodulation. Adv Mater 2014;26:6530–41. 25155610 10.1002/adma.201402105 PMC4269549

[7] Pires IS , Hammond PT, Irvine DJ. Engineering strategies for immunomodulatory cytokine therapies: challenges and clinical progress. Adv Ther 2021;4:2100035. 10.1002/adtp.202100035 PMC8562465 34734110

[8] Franke DD , Shirwan H. IL-2 receptor targeted immunomodulatory biologics: the past, present, and future. CIR 2006;2:187–208.

[9] Thomas TP , Goonewardena SN, Majoros IJ, Kotlyar A, Cao Z, Leroueil PR, Baker Jr JR. Folate-targeted nanoparticles show efficacy in the treatment of inflammatory arthritis. Arthritis Rheum 2011;63:2671–80. 21618461 10.1002/art.30459 PMC3168725

[10] Schweingruber N , Haine A, Tiede K, Karabinskaya A, Brandt JVD, Wust S, Metselaar JM, Gold R, Tuckermann JP, Reichardt HM, Luhder F. Liposomal encapsulation of glucocorticoids alters their mode of action in the treatment of experimental autoimmune encephalomyelitis. J Immunol 2011;187:4310–8. 21918186 10.4049/jimmunol.1101604

[11] Dellacherie MO , Seo BR, Mooney DJ. Macroscale biomaterials strategies for local immunomodulation. Nat Rev Mater 2019;4:379–97.

[12] Leach DG , Young S, Hartgerink JD. Advances in immunotherapy delivery from implantable and injectable biomaterials. Acta Biomater 2019;88:15–31. 30771535 10.1016/j.actbio.2019.02.016 PMC6632081

[13] Hamidi M , Azadi A, Rafiei P. Hydrogel nanoparticles in drug delivery. Adv Drug Deliv Rev 2008;60:1638–49. 18840488 10.1016/j.addr.2008.08.002

[14] Wei W , Zhang Q, Zhou W, Liu Z, Wang Y, Alakpa EV, Ouyang H, Liu H. Immunomodulatory application of engineered hydrogels in regenerative medicine. Appl Mater Today 2019;14:126–36.

[15] Saleh B , Dhaliwal HK, Portillo‐Lara R, Sani ES, Abdi R, Amiji MM, Annabi N. Local immunomodulation using an adhesive hydrogel loaded with miRNA-laden nanoparticles promotes wound healing. Small 2019;15:1902232. 10.1002/smll.201902232 PMC6726510 31328877

[16] Park CG , Hartl CA, Schmid D, Carmona EM, Kim HJ, Goldberg MS. Extended release of perioperative immunotherapy prevents tumor recurrence and eliminates metastases. Sci Transl Med 2018;10:eaar1916. 29563317 10.1126/scitranslmed.aar1916

[17] Ayenehdeh JM , Niknam B, Rasouli S, Hashemi SM, Rahavi H, Rezaei N, Soleimani M, Liaeiha A, Niknam MH, Tajik N. Immunomodulatory and protective effects of adipose tissue-derived mesenchymal stem cells in an allograft islet composite transplantation for experimental autoimmune type 1 diabetes. Immunol Lett 2017;188:21–31. 28506774 10.1016/j.imlet.2017.05.006

[18] Xavier JR , Thakur T, Desai P, Jaiswal MK, Sears N, Cosgriff-Hernandez E, Kaunas R, Gaharwar AK. Bioactive nanoengineered hydrogels for bone tissue engineering: a growth-factor-free approach. ACS Nano 2015;9:3109–18. 25674809 10.1021/nn507488s

[19] Okesola BO , Ni S, Derkus B, Galeano CC, Hasan A, Wu Y, Ramis J, Buttery L, Dawson JI, D’Este M, Oreffo ROC, Eglin D, Sun H, Mata A. Growth-factor free multicomponent nanocomposite hydrogels that stimulate bone formation. Adv Funct Mater 2020;30:1906205.

[20] Wang H , Morales RTT, Cui X, Huang J, Qian W, Tong J, Chen W. A photoresponsive hyaluronan hydrogel nanocomposite for dynamic macrophage immunomodulation. Adv Healthcare Mater 2019;8:1801234. 10.1002/adhm.201801234 PMC6392032 30537061

[21] Cha BH , Shin SR, Leijten J, Li YC, Singh S, Liu JC, Annabi N, Abdi R, Dokmeci MR, Vrana NE, Ghaemmaghami AM, Khademhosseini A. Integrin-mediated interactions control macrophage polarization in 3D hydrogels. Adv Healthcare Mater 2017;6:1700289. 10.1002/adhm.201700289 PMC5677560 28782184

[22] Jones JR. Observing cell response to biomaterials. Mater Today 2006;9:34–43.

[23] Sadtler K , Singh A, Wolf MT, Wang X, Pardoll DM, Elisseeff JH. Design, clinical translation and immunological response of biomaterials in regenerative medicine. Nat Rev Mater 2016;1:1–17.

[24] Okesola BO , Lau HK, Derkus B, Boccorh DK, Wu Y, Wark AW, Kiick KL, Mata A. Covalent co-assembly between resilin-like polypeptide and peptide amphiphile into hydrogels with controlled nanostructure and improved mechanical properties. Biomater Sci 2020;8:846–57. 31793933 10.1039/c9bm01796h PMC7482191

[25] Redondo-Gómez C , Padilla-Lopategui S, Azevedo HS, Mata A. Host–guest-mediated epitope presentation on self-assembled peptide amphiphile hydrogels. ACS Biomater Sci Eng 2020;6:4870–80. 33455284 10.1021/acsbiomaterials.0c00549

[26] Mata A , Hsu L, Capito R, Aparicio C, Henrikson K, Stupp SI. Micropatterning of bioactive self-assembling gels. Soft Matter 2009;5:1228–36. 20047022 10.1039/b819002j PMC2680507

[27] Hedegaard CL , Collin EC, Redondo‐Gómez C, Nguyen LTH, Ng KW, Castrejon-Pita AA, Castrejon-Pita JR, Mata A. Hydrodynamically guided hierarchical self-assembly of peptide–protein bioinks. Adv Funct Mater 2018;28:1703716.

[28] Mitragotri S , Lahann J. Physical approaches to biomaterial design. Nat Mater 2009;8:15–23. 19096389 10.1038/nmat2344 PMC2793340

[29] Sridharan R , Cameron AR, Kelly DJ, Kearney CJ, O’Brien FJ. Biomaterial based modulation of macrophage polarization: a review and suggested design principles. Mater Today 2015;18:313–25.

[30] Discher DE , Janmey P, Wang YL. Tissue cells feel and respond to the stiffness of their substrate. Science 2005;310:1139–43. 16293750 10.1126/science.1116995

[31] Engler AJ , Sen S, Sweeney HL, Discher DE. Matrix elasticity directs stem cell lineage specification. Cell 2006;126:677–89. 16923388 10.1016/j.cell.2006.06.044

[32] Ingavle G , Vaidya A, Kale V. Constructing three-dimensional microenvironments using engineered biomaterials for hematopoietic stem cell expansion. Tissue Eng Part B Rev 2019;25:312–29. 30950320 10.1089/ten.TEB.2018.0286

[33] Amani H , Arzaghi H, Bayandori M, Dezfuli AS, Pazoki-Toroudi H, Shafiee A, Moradi L. Controlling cell behavior through the design of biomaterial surfaces: a focus on surface modification techniques. Adv Mater Interfaces 2019;6:1900572.

[34] Derkus B , Okesola BO, Barrett DW, D’Este M, Chowdhury TT, Eglin D, Mata A. Multicomponent hydrogels for the formation of vascularized bone-like constructs in vitro. Acta Biomater 2020;109:82–94. 32311533 10.1016/j.actbio.2020.03.025

[35] Hedegaard CL , Redondo-Gómez C, Tan BY, Loessner D, Mata A. Peptide-protein coassembling matrices as a biomimetic 3D model of ovarian cancer. Sci Adv 2020;6:eabb3298. 33008910 10.1126/sciadv.abb3298 PMC7852381

[36] Brassard JA , Nikolaev M, Hübscher T, Hofer M, Lutolf MP. Recapitulating macro-scale tissue self-organization through organoid bioprinting. Nat Mater 2021;20:22–8. 32958879 10.1038/s41563-020-00803-5

[37] Bridges AW , Singh N, Burns KL, Babansee JE, Lyon LA, Garcia AJ. Reduced acute inflammatory responses to microgel conformal coatings. Biomaterials 2008;29:4605–15. 18804859 10.1016/j.biomaterials.2008.08.015 PMC2585524

[38] Vishwakarma A , Bhise NS, Evangelista MB, Rouwkema J, Dokmeci MR, Ghaemmaghami AM, Vrana NE, Khademhosseini A. Engineering immunomodulatory biomaterials to tune the inflammatory response. Trends Biotechnol 2016;34:470–82. 27138899 10.1016/j.tibtech.2016.03.009

[39] Caliari SR , Vega SL, Kwon M, Soulas EM, Burdick JA. Dimensionality and spreading influence MSC YAP/TAZ signaling in hydrogel environments. Biomaterials 2016;103:314–23. 27429252 10.1016/j.biomaterials.2016.06.061 PMC4963302

[40] Wen JH , Vincent LG, Fuhrmann A, Choi YS, Hribar KC, Taylor-Weiner H, Chen S, Engler AJ. Interplay of matrix stiffness and protein tethering in stem cell differentiation. Nat Mater 2014;13:979–87. 25108614 10.1038/nmat4051 PMC4172528

[41] Smith AG , Din A, Denyer M, Crowther NJ, Eagland D, Vowden K, Vowden P, Britland ST. Microengineered surface topography facilitates cell grafting from a prototype hydrogel wound dressing with antibacterial capability. Biotechnol Prog 2006;22:1407–15. 17022681 10.1021/bp060192n

[42] You J , Raghunathan VK, Son KJ, Patel D, Haque A, Murphy CJ, Revzin A. Impact of nanotopography, heparin hydrogel microstructures, and encapsulated fibroblasts on phenotype of primary hepatocytes. ACS Appl Mater Interfaces 2015;7:12299–308. 25247391 10.1021/am504614e PMC4372509

[43] Singh S , Awuah D, Rostam HM, Emes RD, Kandola NK, Onion D, Htwe SS, Rajchagool B, Cha BH, Kim D, Tighe PJ, Vrana NE, Khademhosseini A, Ghaemmaghami A. Unbiased analysis of the impact of micropatterned biomaterials on macrophage behavior provides insights beyond predefined polarization states. ACS Biomater Sci Eng 2017;3:969–78. 33429569 10.1021/acsbiomaterials.7b00104

[44] Xu Z , Hwang D-G, Bartlett MD, Jiang S, Bratlie KM. Alter macrophage adhesion and modulate their response on hydrophobically modified hydrogels. *Biochem Eng J* 2021;**165**:107821.

[45] Schneider GB , English A, Abraham M, Zaharias R, Stanford C, Keller J. The effect of hydrogel charge density on cell attachment. Biomaterials 2004;25:3023–8. 14967535 10.1016/j.biomaterials.2003.09.084

[46] Qiu P , Li M, Chen K, Fang B, Chen P, Tang Z, Lin X, Fan S. Periosteal matrix-derived hydrogel promotes bone repair through an early immune regulation coupled with enhanced angio-and osteogenesis. Biomaterials 2020;227:119552. 31670079 10.1016/j.biomaterials.2019.119552

[47] Veiseh O , Vegas AJ. Domesticating the foreign body response: recent advances and applications. Adv Drug Deliv Rev 2019;144:148–61. 31491445 10.1016/j.addr.2019.08.010 PMC6774350

[48] Franz S , Rammelt S, Scharnweber D, Simon JC. Immune responses to implants–a review of the implications for the design of immunomodulatory biomaterials. Biomaterials 2011;32:6692–709. 21715002 10.1016/j.biomaterials.2011.05.078

[49] Schmaier AH. Contact activation: a revision. Thromb Haemost 1997;78:101–7. 9198136

[50] Sperling C , Fischer M, Maitz MF, Werner C. Blood coagulation on biomaterials requires the combination of distinct activation processes. Biomaterials 2009;30:4447–56. 19535136 10.1016/j.biomaterials.2009.05.044

[51] Heemskerk JW , Bevers EM, Lindhout T. Platelet activation and blood coagulation. Thromb Haemost 2002;88:186–93. 12195687

[52] Savage B , Bottini E, Ruggeri ZM. Interaction of integrin αIIbβ3 with multiple fibrinogen domains during platelet adhesion. J Biol Chem 1995;270:28812–7. 7499405 10.1074/jbc.270.48.28812

[53] Rodrigues SN , Gonçalves IC, Martins M, Barbosa MA, Ratner BD. Fibrinogen adsorption, platelet adhesion and activation on mixed hydroxyl-/methyl-terminated self-assembled monolayers. Biomaterials 2006;27:5357–67. 16842847 10.1016/j.biomaterials.2006.06.010

[54] Fischer M , Sperling C, Tengvall P, Werner C. The ability of surface characteristics of materials to trigger leukocyte tissue factor expression. Biomaterials 2010;31:2498–507. 20035991 10.1016/j.biomaterials.2009.12.016

[55] Diegelmann R , Evans M. Wound healing: an overview of acute, fibrotic and delayed healing. Front Biosci 2004;9:283–9. 14766366 10.2741/1184

[56] Lambris JD , Ekdahl KN, Ricklin D, Nilsson B *Immune Responses to Biosurfaces*. Switzerland:Springer, 2015.

[57] Kobayashi SD , Voyich JM, Burlak C, DeLeo FR. Neutrophils in the innate immune response. Arch Immunol Ther Exp (Warsz) 2005;53:505–17. 16407783

[58] Nimeri G , Öhman L, Elwing H, Wettero J, Bengtsson T. The influence of plasma proteins and platelets on oxygen radical production and F-actin distribution in neutrophils adhering to polymer surfaces. Biomaterials 2002;23:1785–95. 11950049 10.1016/s0142-9612(01)00305-2

[59] Aimetti AA , Tibbitt MW, Anseth KS. Human neutrophil elastase responsive delivery from poly (ethylene glycol) hydrogels. Biomacromolecules 2009;10:1484–9. 19408953 10.1021/bm9000926 PMC2699883

[60] Kolaczkowska E , Kubes P. Neutrophil recruitment and function in health and inflammation. Nat Rev Immunol 2013;13:159–75. 23435331 10.1038/nri3399

[61] Brinkmann V , Reichard U, Goosmann C, Fauler B, Uhlemann Y, Weiss DS, Weinrauch Y, Zychlinsky A. Neutrophil extracellular traps kill bacteria. Science 2004;303:1532–5. 15001782 10.1126/science.1092385

[62] Hahn J , Schauer C, Czegley C, Kling L, Petru L, Schmid B, Weidner D, Reinwald C, Biermann MHC, Blunder S, Ernst J, Lesner A, Bauerle T, Palmisano R, Christiansen S, Herrmann M, Bozec A, Gruber R, Schett G, Hoffmann MH. Aggregated neutrophil extracellular traps resolve inflammation by proteolysis of cytokines and chemokines and protection from antiproteases. Faseb J 2019;33:1401–14. 30130433 10.1096/fj.201800752R PMC6355082

[63] Schauer C , Janko C, Munoz LE, Zhao Y, Kienhofer D, Frey B, Lell M, Manger B, Rech J., Naschberger E, Holmdahl R, Krenn V, Harrer T, Jeremic I, Bilyy R, Schett G, Hoffmann M, Herrmann M. Aggregated neutrophil extracellular traps limit inflammation by degrading cytokines and chemokines. Nat Med 2014;20:511–7. 24784231 10.1038/nm.3547

[64] Yamashiro S , Kamohara H, Wang JM, Yang D, Gong WH, Yoshimura T. Phenotypic and functional change of cytokine-activated neutrophils: inflammatory neutrophils are heterogeneous and enhance adaptive immune responses. J Leukoc Biol 2001;69:698–704. 11358976

[65] Jones JA , Chang DT, Meyerson H, Colton E, Kwon IK, Matsuda T, Anderson JM. Proteomic analysis and quantification of cytokines and chemokines from biomaterial surface-adherent macrophages and foreign body giant cells. J Biomed Mater Res A 2007;83:585–96. 17503526 10.1002/jbm.a.31221

[66] Broughton G I , Janis JE, Attinger CE. The basic science of wound healing. Plast Reconstr Surg 2006;117:12S–34S. 16799372 10.1097/01.prs.0000225430.42531.c2

[67] Mantovani A , Sica A, Sozzani S, Allavena P, Vecchi A, Locati M. The chemokine system in diverse forms of macrophage activation and polarization. Trends Immunol 2004;25:677–86. 15530839 10.1016/j.it.2004.09.015

[68] Mosser DM , Edwards JP. Exploring the full spectrum of macrophage activation. Nat Rev Immunol 2008;8:958–69. 19029990 10.1038/nri2448 PMC2724991

[69] Martinez FO , Sica A, Mantovani A, Locati M. Macrophage activation and polarization. Front Biosci 2008;13:453–61. 17981560 10.2741/2692

[70] Stein M , Keshav S, Harris N, Gordon S. Interleukin 4 potently enhances murine macrophage mannose receptor activity: a marker of alternative immunologic macrophage activation. J Exp Med 1992;176:287–92. 1613462 10.1084/jem.176.1.287 PMC2119288

[71] Wynn TA , Vannella KM. Macrophages in tissue repair, regeneration, and fibrosis. Immunity 2016;44:450–62. 26982353 10.1016/j.immuni.2016.02.015 PMC4794754

[72] Anderson JM. Biological responses to materials. Annu Rev Mater Res 2001;31:81–110.

[73] Veiseh O , Doloff JC, Ma M, Vegas AJ, Tam HH, Bader AR, Li J, Langan E, Wyckoff J, Loo WS, Jhunjhunwala S, Chiu A, Siebert S, Tang K, Hollister-Lock J, Aresta-Dasilva S, Bochenek M, Mendoza-Elias J, Wang Y, Qi M, Lavin DM, Chen M, Dholakia N, Thakrar R, Lacik I, Weir GC, Oberholzer J, Greiner DL, Langer R, Anderson DG. Size-and shape-dependent foreign body immune response to materials implanted in rodents and non-human primates. Nat Mater 2015;14:643–51. 25985456 10.1038/nmat4290 PMC4477281

[74] Vassey MJ , Figueredo GP, Scurr DJ, Vasilevich AS, Vermeulen S, Carlier A, Luckett J, Beijer NRM, Williams P, Winkler DA, Boer JD, Ghaemmaghami AM, Alexander MR. Immune modulation by design: using topography to control human monocyte attachment and macrophage differentiation. Adv Sci (Weinh) 2020;7:1903392. 32537404 10.1002/advs.201903392 PMC7284204

[75] Stout RD , Jiang C, Matta B, Tietzel I, Watkins SK, Suttles J. Macrophages sequentially change their functional phenotype in response to changes in microenvironmental influences. J Immunol 2005;175:342–9. 15972667 10.4049/jimmunol.175.1.342

[76] Franz S , Hoeve MA, Wickert S, Janko C, Dransfield I. Clearance of apo Nph induces an immunosuppressive response in pro-inflammatory type-1 and anti-inflammatory type-2 MΦ: brief definite report. Autoimmunity 2009;42:275–7. 19811275 10.1080/08916930902828080

[77] Frick J-S , Grünebach F, Autenrieth IB. Immunomodulation by semi-mature dendritic cells: a novel role of Toll-like receptors and interleukin-6. Int J Med Microbiol 2010;300:19–24. 19781988 10.1016/j.ijmm.2009.08.010

[78] Acharya AP , Dolgova NV, Clare-Salzler MJ, Keselowsky BG. Differential levels of dendritic cell maturation on different biomaterials used in combination products. J Biomed Mater Res A 2005;74:503–10. 16158496 10.1002/jbm.a.30429

[79] Yoshida M , Babensee JE. Differential effects of agarose and poly (lactic-co-glycolic acid) on dendritic cell maturation. J Biomed Mater Res A 2006;79:393–408. 16886225 10.1002/jbm.a.30798

[80] Acharya AP , Dolgova NV, Clare-Salzler MJ, Keselowsky BG. Adhesive substrate-modulation of adaptive immune responses. Biomaterials 2008;29:4736–50. 18829103 10.1016/j.biomaterials.2008.08.040

[81] Yoshida M , Babensee JE. Poly (lactic-co-glycolic acid) enhances maturation of human monocyte-derived dendritic cells. J Biomed Mater Res A 2004;71:45–54. 15368253 10.1002/jbm.a.30131

[82] Wang H , Luo Z, Wang Y, He T, Yang C, Ren C, Ma L, Gong C, Li X, Yang Z. Enzyme-catalyzed formation of supramolecular hydrogels as promising vaccine adjuvants. Adv Funct Mater 2016;26:1822–9.

[83] Luo Z , Wu Q, Yang C, Wang H, He T, Wang Y, Wang Z, Chen H, Li X, Gong C, Yang Z. A powerful CD8+ T-cell stimulating D-tetra-peptide hydrogel as a very promising vaccine adjuvant. Adv Mater 2017;29:1601776. 10.1002/adma.201601776 27859662

[84] Brodbeck WG , MacEwan M, Colton E, Meyerson H, Anderson JM. Lymphocytes and the foreign body response: lymphocyte enhancement of macrophage adhesion and fusion. J Biomed Mater Res A 2005;74:222–9. 15948198 10.1002/jbm.a.30313

[85] Sadtler K , Estrellas K, Allen BW, Wolf MT, Fan H, Tam AJ, Patel CH, Luber BS, Wang H, Wagner KR, Powell JD, Housseau F, Pardoll DM, Elisseeff J. Developing a pro-regenerative biomaterial scaffold microenvironment requires T helper 2 cells. Science 2016;352:366–70. 27081073 10.1126/science.aad9272 PMC4866509

[86] Chang DT , Colton E, Anderson JM. Paracrine and juxtacrine lymphocyte enhancement of adherent macrophage and foreign body giant cell activation. J Biomed Mater Res A 2009;89:490–8. 18437695 10.1002/jbm.a.31981 PMC3864690

[87] Chang DT , Jones JA, Meyerson H, Colton E, Kwon IK, Matsuda T, Anderson JM. Lymphocyte/macrophage interactions: biomaterial surface-dependent cytokine, chemokine, and matrix protein production. J Biomed Mater Res A 2008;87:676–87. 18200554 10.1002/jbm.a.31630 PMC3867010

[88] Marques AP , Reis RL, Hunt JA. Cytokine secretion from mononuclear cells cultured in vitro with starch-based polymers and poly-L-lactide. J Biomed Mater Res A 2004;71:419–29. 15472922 10.1002/jbm.a.30155

[89] Young KD. The selective value of bacterial shape. Microbiol Mol Biol Rev 2006;70:660–703. 16959965 10.1128/MMBR.00001-06 PMC1594593

[90] Conklin MW , Eickhoff JC, Riching KM, Pehlke CA, Eliceiri KW, Provenzano PP, Friedl A, Keely PJ. Aligned collagen is a prognostic signature for survival in human breast carcinoma. Am J Pathol 2011;178:1221–32. 21356373 10.1016/j.ajpath.2010.11.076 PMC3070581

[91] Mouw JK , Yui Y, Damiano L, Bainer RO, Lakins JN, Acerbi I, Ou G, Wijekoon AC, Levental KR, Gilbert PM, Hwang ES, Chen YY, Weaver VM. Tissue mechanics modulate microRNA-dependent PTEN expression to regulate malignant progression. Nat Med 2014;20:360–7. 24633304 10.1038/nm.3497 PMC3981899

[92] Yamahashi Y , Cavnar PJ, Hind LE, Berthier E, Bennin DA, Beede D, Huttenlocher A. Integrin associated proteins differentially regulate neutrophil polarity and directed migration in 2D and 3D. Biomed Microdevices 2015;17:100. 26354879 10.1007/s10544-015-9998-x PMC4678772

[93] Friedemann M , Kalbitzer L, Franz S, Moeller S, Schnabelrauch M, Simon JC, Pompe T, Franke K. Instructing human macrophage polarization by stiffness and glycosaminoglycan functionalization in 3D collagen networks. Adv Healthcare Mater 2017;6:1600967. 10.1002/adhm.201600967 28135049

[94] Bhattacharya A , Agarwal M, Mukherjee R, Sen P, Sinha DK. 3D micro-environment regulates NF-κβ dependent adhesion to induce monocyte differentiation. Cell Death Dis 2018;9:1–16. 30206232 10.1038/s41419-018-0993-z PMC6133927

[95] Kim M , Lee S, Ki CS. Cellular behavior of RAW264. 7 cells in 3D poly (ethylene glycol) hydrogel niches. ACS Biomater Sci Eng 2019;5:922–32. 33405849 10.1021/acsbiomaterials.8b01150

[96] Choi YS , Jeong E, Lee JS, Kim SK, Jo SH, Kim YG, Sung HJ, Cho SW, Jin Y. Immunomodulatory scaffolds derived from lymph node extracellular matrices. ACS Appl Mater Interfaces 2021;13:14037–49. 33745275 10.1021/acsami.1c02542

[97] Gosselin EA , Eppler HB, Bromberg JS, Jewell CM. Designing natural and synthetic immune tissues. Nat Mater 2018;17:484–98. 29784994 10.1038/s41563-018-0077-6 PMC6283404

[98] Purwada A , Jaiswal MK, Ahn H, Nojima T, Kitamura D, Gaharwar AK, Cerchietti L, Singh A. Ex vivo engineered immune organoids for controlled germinal center reactions. Biomaterials 2015;63:24–34. 26072995 10.1016/j.biomaterials.2015.06.002 PMC4490011

[99] Purwada A , Singh A. Immuno-engineered organoids for regulating the kinetics of B-cell development and antibody production. Nat Protoc 2017;12:168–82. 28005068 10.1038/nprot.2016.157 PMC6355337

[100] Nichols JE , Cortiella J, Lee J, Niles JA, Cuddihy M, Wang S, Bielitzki J, Cantu A, Mlcak R, Valdivia E, Yancy R, McClure ML, Kotov NA. In vitro analog of human bone marrow from 3D scaffolds with biomimetic inverted colloidal crystal geometry. Biomaterials 2009;30:1071–9. 19042018 10.1016/j.biomaterials.2008.10.041 PMC2650812

[101] Baumgart F. Stiffness-an unknown world of mechanical science? Injury 2000;31:14–23. 10853758

[102] Guimarães CF , Gasperini L, Marques AP, Reis RL. The stiffness of living tissues and its implications for tissue engineering. Nat Rev Mater 2020;5:351–70.

[103] Oakes PW , Patel DC, Morin NA, Zitterbart DP, Fabry B, Reichner JS, Tang JX. Neutrophil morphology and migration are affected by substrate elasticity. Blood 2009;114:1387–95. 19491394 10.1182/blood-2008-11-191445 PMC2727411

[104] Devreotes PN , Zigmond SH. Chemotaxis in eukaryotic cells: a focus on leukocytes and Dictyostelium. Annu Rev Cell Biol 1988;4:649–86. 2848555 10.1146/annurev.cb.04.110188.003245

[105] Jannat RA , Dembo M, Hammer DA. Traction forces of neutrophils migrating on compliant substrates. Biophys J 2011;101:575–84. 21806925 10.1016/j.bpj.2011.05.040 PMC3145281

[106] Blakney AK , Swartzlander MD, Bryant SJ. The effects of substrate stiffness on the in vitro activation of macrophages and in vivo host response to poly (ethylene glycol)-based hydrogels. J Biomed Mater Res A 2012;100:1375–86. 22407522 10.1002/jbm.a.34104 PMC3339197

[107] Sridharan R , Cavanagh B, Cameron AR, Kelly DJ, O’Brien FJ. Material stiffness influences the polarization state, function and migration mode of macrophages. Acta Biomater 2019;89:47–59. 30826478 10.1016/j.actbio.2019.02.048

[108] He X-T , Wu R-X, Xu X-Y, Wang J, Yin Y, Chen F-M. Macrophage involvement affects matrix stiffness-related influences on cell osteogenesis under three-dimensional culture conditions. Acta Biomater 2018;71:132–47. 29462712 10.1016/j.actbio.2018.02.015

[109] Zhuang Z , Zhang Y, Sun S, Li Q, Chen K, An C, Wang L, Beucken JJJPVD, Wang H. Control of matrix stiffness using methacrylate–gelatin hydrogels for a macrophage-mediated inflammatory response. ACS Biomater Sci Eng 2020;6:3091–102. 33463297 10.1021/acsbiomaterials.0c00295

[110] Li Z , Bratlie KM. How cross-linking mechanisms of methacrylated gellan gum hydrogels alter macrophage phenotype. ACS Appl Bio Mater 2019;2:217–25. 10.1021/acsabm.8b00562 35016344

[111] Judokusumo E , Tabdanov E, Kumari S, Dustin ML, Kam LC. Mechanosensing in T lymphocyte activation. Biophys J 2012;102:L5–L7. 22339876 10.1016/j.bpj.2011.12.011 PMC3260692

[112] Saitakis M , Dogniaux S, Goudot C, Bufi N, Asnacios S, Maurin M, Randriamampita C, Asnacios A, Hivroz C. Different TCR-induced T lymphocyte responses are potentiated by stiffness with variable sensitivity. Elife 2017;6:e23190. 28594327 10.7554/eLife.23190 PMC5464771

[113] Pearce EL , Poffenberger MC, Chang C-H, Jones RG. Fueling immunity: insights into metabolism and lymphocyte function. Science 2013;342:1242454. 24115444 10.1126/science.1242454 PMC4486656

[114] Wan Z , Zhang S, Fan Y, Liu K, Du F, Davey AM, Zhang H, Han W, Xiong C, Liu W. B cell activation is regulated by the stiffness properties of the substrate presenting the antigens. J Immunol 2013;190:4661–75. 23554309 10.4049/jimmunol.1202976

[115] Hirsch S , Guo J, Reiter R, Papazoglou S, Kroencke T, Braun J, Sack I. MR elastography of the liver and the spleen using a piezoelectric driver, single-shot wave-field acquisition, and multifrequency dual parameter reconstruction. Magn Reson Med 2014;71:267–77. 23413115 10.1002/mrm.24674

[116] He X-T , Li X, Xia Y, Yin Y, Wu R-X, Sun H-H, Chen F-M. Building capacity for macrophage modulation and stem cell recruitment in high-stiffness hydrogels for complex periodontal regeneration: experimental studies in vitro and in rats. Acta Biomater 2019;88:162–80. 30735811 10.1016/j.actbio.2019.02.004

[117] Haeger A , Krause M, Wolf K, Friedl P. Cell jamming: collective invasion of mesenchymal tumor cells imposed by tissue confinement. Biochim Biophys Acta 2014;1840:2386–95. 24721714 10.1016/j.bbagen.2014.03.020

[118] Singh A , Suri S, Roy K. In-situ crosslinking hydrogels for combinatorial delivery of chemokines and siRNA–DNA carrying microparticles to dendritic cells. Biomaterials 2009;30:5187–200. 19560815 10.1016/j.biomaterials.2009.06.001 PMC2818033

[119] Verbeke CS , Mooney DJ. Injectable, pore-forming hydrogels for in vivo enrichment of immature dendritic cells. Adv Healthc Mater 2015;4:2677–87. 26474318 10.1002/adhm.201500618 PMC4715727

[120] Liu Y , Wang L, Kikuiri T, Akiyama K, Chen C, Xu X, Yang R, Chen W, Wang S, Shi S. Mesenchymal stem cell–based tissue regeneration is governed by recipient T lymphocytes via IFN-γ and TNF-α. Nat Med 2011;17:1594–601. 22101767 10.1038/nm.2542 PMC3233650

[121] Moshaverinia A , Chen C, Xu X, Ansari S, Zadeh HH, Schricker SR, Paine ML, Moradian-Oldak J, Khademhosseini A, Snead ML, Shi S. Regulation of the stem cell–host immune system interplay using hydrogel coencapsulation system with an anti-inflammatory drug. Adv Funct Mater 2015;25:2296–307. 26120294 10.1002/adfm.201500055 PMC4478611

[122] Sussman EM , Halpin MC, Muster J, Moon RT, Ratner BD. Porous implants modulate healing and induce shifts in local macrophage polarization in the foreign body reaction. Ann Biomed Eng 2014;42:1508–16. 24248559 10.1007/s10439-013-0933-0

[123] Madden LR , Mortisen DJ, Sussman EM, Dupras SK, Fugate JA, Cuy JL, Hauch KD, Laflamme MA, Murry CE, Ratner BD. Proangiogenic scaffolds as functional templates for cardiac tissue engineering. Proc Natl Acad Sci USA 2010;107:15211–6. 20696917 10.1073/pnas.1006442107 PMC2930533

[124] Yin Y , He XT, Wang J, Wu RX, Xu XY, Hong YL, Tian BM, Chen FM. Pore size-mediated macrophage M1-to-M2 transition influences new vessel formation within the compartment of a scaffold. Appl Mater Today 2020;18:100466.

[125] Camarero‐Espinosa S , Carlos‐Oliveira M, Liu H, Mano JF, Bouvy N, Moroni L. 3D printed dual-porosity scaffolds: the combined effect of stiffness and porosity in the modulation of macrophage polarization. Adv Healthcare Mater 2022;11:2101415. 10.1002/adhm.202101415 PMC11468864 34719861

[126] Li X , Cho B, Martin R, Seu M, Zhang C, Zhou Z, Choi JS, Jiang X, Chen L, Walia G, Yan J, Callanan M, Liu H, Colbert K, Morrissette-Mcalmon J, Grayson W, Reddy S, Sacks JM, Mao HQ. Nanofiber-hydrogel composite–mediated angiogenesis for soft tissue reconstruction. Sci Transl Med 2019;11:eaau6210. 31043572 10.1126/scitranslmed.aau6210 PMC12989237

[127] Takahashi M , Heo Y, Shibata H, Satou H, Kawanishi T, Okitsu T, Takeuchi S. Nano-patterned hydrogel reduced inflammatory effects in subcutaneous tissue. In: *IEEE 25th International Conference on Micro Electro Mechanical Systems (MEMS)*, 2012. pp. 973–976. IEEE.

[128] Xing R , Li S, Zhang N, Shen G, Mohwald H, Yan X. Self-assembled injectable peptide hydrogels capable of triggering antitumor immune response. Biomacromolecules 2017;18:3514–23. 28721731 10.1021/acs.biomac.7b00787

[129] Hersel U , Dahmen C, Kessler H. RGD modified polymers: biomaterials for stimulated cell adhesion and beyond. Biomaterials 2003;24:4385–415. 12922151 10.1016/s0142-9612(03)00343-0

[130] Taipale J , Keski-Oja J. Growth factors in the extracellular matrix. Faseb J 1997;11:51–9. 9034166 10.1096/fasebj.11.1.9034166

[131] Ceccarelli B , Grohovaz F, Hurlbut W. Freeze-fracture studies of frog neuromuscular junctions during intense release of neurotransmitter. II. Effects of electrical stimulation and high potassium. J Cell Biol 1979;81:178–92. 39080 10.1083/jcb.81.1.178 PMC2111526

[132] He J , Chen G, Liu M, Xu Z, Chen H, Yang L, Lv Y. Scaffold strategies for modulating immune microenvironment during bone regeneration. Mater Sci Eng C 2020;108:110411. 10.1016/j.msec.2019.110411 31923946

[133] Rostam HM , Singh S, Salazar F, Magennis P, Hook A, Singh T, Vrana VE, Alexander MR, Ghaemmaghami AM. The impact of surface chemistry modification on macrophage polarisation. Immunobiology 2016;221:1237–46. 27349596 10.1016/j.imbio.2016.06.010

[134] Lynn AD , Kyriakides TR, Bryant SJ. Characterization of the in vitro macrophage response and in vivo host response to poly (ethylene glycol)-based hydrogels. J Biomed Mater Res A 2010;93:941–53. 19708075 10.1002/jbm.a.32595

[135] Tangpasuthadol V , Pongchaisirikul N, Hoven VP. Surface modification of chitosan films: effects of hydrophobicity on protein adsorption. Carbohydr Res 2003;338:937–42. 12681917 10.1016/s0008-6215(03)00038-7

[136] da Silva Domingues JF , Roest S, Wang Y, Mei HCVD, Libera M, Kooten TGV, Busscher HJ. Macrophage phagocytic activity toward adhering staphylococci on cationic and patterned hydrogel coatings versus common biomaterials. Acta Biomater 2015;18:1–8. 25752975 10.1016/j.actbio.2015.02.028

[137] Weaver JC , Astumian RD. The response of living cells to very weak electric fields: the thermal noise limit. Science 1990;247:459–62. 2300806 10.1126/science.2300806

[138] Burr HS , Northrop FSC. Evidence for the existence of an electro-dynamic field in living organisms. Proc Natl Acad Sci USA 1939;25:284–8. 16577899 10.1073/pnas.25.6.284 PMC1077770

[139] Zhao M , Song B, Pu J, Wada T, Reid B, Tai G, Wang F, Guo A, Walczysko P, Gu Y, Sasaki T, Suzuki A, Forrester JV, Bourne HR, Devreotes PN, McCaig CD, Penninger JM. Electrical signals control wound healing through phosphatidylinositol-3-OH kinase-γ and PTEN. Nature 2006;442:457–60. 16871217 10.1038/nature04925

[140] Huttenlocher A , Horwitz AR. Wound healing with electric potential. N Engl J Med 2007;356:303–4. 17229960 10.1056/NEJMcibr066496

[141] Gessner A , Lieske A, Paulke BR, Muller RH. Influence of surface charge density on protein adsorption on polymeric nanoparticles: analysis by two-dimensional electrophoresis. Eur J Pharm Biopharm 2002;54:165–70. 12191688 10.1016/s0939-6411(02)00081-4

[142] Samavedi S , Whittington AR, Goldstein AS. Calcium phosphate ceramics in bone tissue engineering: a review of properties and their influence on cell behavior. Acta Biomater 2013;9:8037–45. 23791671 10.1016/j.actbio.2013.06.014

[143] Smetana Jr , Vacik K, Součková J, Krčová D, Šulc ZJ. The influence of hydrogel functional groups on cell behavior. J Biomed Mater Res 1990;24:463–70. 2189879 10.1002/jbm.820240405

[144] Zhang J , Zhu Y, Song J, Yang J, Pan C, Xu T, Zhang L. Novel balanced charged alginate/PEI polyelectrolyte hydrogel that resists foreign-body reaction. ACS Appl Mater Interfaces 2018;10:6879–86. 29393622 10.1021/acsami.7b17670

[145] Lopez-Silva TL , Leach DG, Azares A, Li IC, Woodside DG, Hartgerink JD. Chemical functionality of multidomain peptide hydrogels governs early host immune response. Biomaterials 2020;231:119667. 31855625 10.1016/j.biomaterials.2019.119667 PMC7049098

[146] Taraballi F , Sushnitha M, Tsao C, Bauza G, Liverani C, Shi A, Tasciotti E. Biomimetic tissue engineering: tuning the immune and inflammatory response to implantable biomaterials. Adv Healthcare Mater 2018;7:1800490. 10.1002/adhm.201800490 29995315

[147] Sicari BM , Dziki JL, Siu BF, Medberry CJ, Dearth CL, Badylak SF. The promotion of a constructive macrophage phenotype by solubilized extracellular matrix. Biomaterials 2014;35:8605–12. 25043569 10.1016/j.biomaterials.2014.06.060

[148] Dziki JL , Wang DS, Pineda C, Sicari BM, Rausch T, Badylak SF. Solubilized extracellular matrix bioscaffolds derived from diverse source tissues differentially influence macrophage phenotype. J Biomed Mater Res 2017;105:138–47. 10.1002/jbm.a.35894 27601305

[149] Lin T , Liu S, Chen S, Qiu S, Rao Z, Liu J, Zhu S, Yan L, Mao H, Zhu Q, Quan D, Liu X. Hydrogel derived from porcine decellularized nerve tissue as a promising biomaterial for repairing peripheral nerve defects. Acta Biomater 2018;73:326–38. 29649641 10.1016/j.actbio.2018.04.001

[150] Rowley AT , Nagalla RR, Wang SW, Liu WF. Extracellular matrix-based strategies for immunomodulatory biomaterials engineering. Adv Healthcare Mater 2019;8:1801578. 10.1002/adhm.201801578 PMC7568845 30714328

[151] Donaldson AR , Tanase CE, Awuah D, Bathrinarayanan PV, Hall L, Nikkhah M, Khademhosseini A, Rose F, Alexander C, Ghaemmaghami AM. Photocrosslinkable gelatin hydrogels modulate the production of the major pro-inflammatory cytokine, TNF-α, by human mononuclear cells. Front Bioeng Biotechnol 2018;6:116. 30283776 10.3389/fbioe.2018.00116 PMC6156527

[152] Hsieh JY , Smith TD, Meli VS, Tran TN, Botvinick EL, Liu WF. Differential regulation of macrophage inflammatory activation by fibrin and fibrinogen. Acta Biomater 2017;47:14–24. 27662809 10.1016/j.actbio.2016.09.024 PMC5426227

[153] Price RD , Myers S, Leigh IM, Navsaria HA. The role of hyaluronic acid in wound healing. Am J Clin Dermatol 2005;6:393–402. 16343027 10.2165/00128071-200506060-00006

[154] Pierschbacher MD , Ruoslahti E. Cell attachment activity of fibronectin can be duplicated by small synthetic fragments of the molecule. Nature 1984;309:30–3. 6325925 10.1038/309030a0

[155] Lynn AD , Bryant SJ. Phenotypic changes in bone marrow-derived murine macrophages cultured on PEG-based hydrogels activated or not by lipopolysaccharide. Acta Biomater 2011;7:123–32. 20674808 10.1016/j.actbio.2010.07.033 PMC2967672

[156] Schiffmann E , Corcoran BA, Wahl SM. N-formylmethionyl peptides as chemoattractants for leucocytes. Proc Natl Acad Sci USA 1975;72:1059–62. 1093163 10.1073/pnas.72.3.1059 PMC432465

[157] Showell H , Freer RJ, Zigmond SH, Schiffmann E, Aswanikumar S, Corcoran B, Becker EL. The structure-activity relations of synthetic peptides as chemotactic factors and inducers of lysosomal secretion for neutrophils. J Exp Med 1976;143:1154–69. 1262785 10.1084/jem.143.5.1154 PMC2190180

[158] Thota CK , Berger AA, Elomaa L, Nie C, Böttcher C, Koksch B. Coassembly generates peptide hydrogel with wound dressing material properties. ACS Omega 2020;5:8557–63. 32337417 10.1021/acsomega.9b04371 PMC7178367

[159] Zhao F , Li J, Zhou N, Sakai J, Gao Y, Shi J, Goldman B, Browdy HM, Luo HR, Xu B. De novo chemoattractants form supramolecular hydrogels for immunomodulating neutrophils in vivo. Bioconjug Chem 2014;25:2116–22. 25398017 10.1021/bc5004923 PMC4275169

[160] Hemmi H , Takeuchi O, Kawai T, Kaisho T, Sato S, Sanjo H, Matsumoto K, Hoshino K, Wagner H, Takeda K, Akira S. A toll-like receptor recognizes bacterial DNA. Nature 2000;408:740–5. 11130078 10.1038/35047123

[161] Nishikawa M , Mizuno Y, Mohri K, Matsuoka N, Rattanakiat S, Takahashi Y, Funabashi H, Luo D, Takakura Y. Biodegradable CpG DNA hydrogels for sustained delivery of doxorubicin and immunostimulatory signals in tumor-bearing mice. Biomaterials 2011;32:488–94. 20932569 10.1016/j.biomaterials.2010.09.013

[162] Nishikawa M , Ogawa K, Umeki Y, Mohri K, Kawasaki Y, Watanabe H, Takahashi N, Kusuki E, Takahashi R, Takahashi Y, Takakura Y. Injectable, self-gelling, biodegradable, and immunomodulatory DNA hydrogel for antigen delivery. J Control Release 2014;180:25–32. 24530618 10.1016/j.jconrel.2014.02.001

[163] Nishida Y , Ohtsuki S, Araie Y, Umeki Y, Endo M, Emura T, Hidaka K, Sugiyama H, Takahashi Y, Takakura Y, Nishikawa M. Self-assembling DNA hydrogel-based delivery of immunoinhibitory nucleic acids to immune cells. Nanomedicine 2016;12:123–30. 26364795 10.1016/j.nano.2015.08.004

[164] Ward WK. A review of the foreign-body response to subcutaneously-implanted devices: the role of macrophages and cytokines in biofouling and fibrosis. J Diabetes Sci Technol 2008;2:768–77. 19885259 10.1177/193229680800200504 PMC2769792

[165] Taskin MB , Tylek T, Blum C, Böhm C, Wiesbeck C, Groll J. Inducing immunomodulatory effects on human macrophages by multifunctional NCO-sP(EO-stat-PO)/gelatin hydrogel nanofibers. ACS Biomater Sci Eng 2021;7:3166–78. 34114792 10.1021/acsbiomaterials.1c00232

[166] Zhang L , Cao Z, Bai T, Carr L, Ella-Menye JR, Irvin C, Ratner BD, Jiang S. Zwitterionic hydrogels implanted in mice resist the foreign-body reaction. Nat Biotechnol 2013;31:553–6. 23666011 10.1038/nbt.2580

[167] Vegas AJ , Veiseh O, Doloff JC, Ma M, Tam HH, Bratlie K, Li J, Bader AR, Langan E, Olejnik K, Fenton P, Kang JW, Hollister-Locke J, Bochenek MA, Chiu A, Siebert S, Tang K, Jhunjhunwala S, Aresta-Dasilva S, Dholakia N, Thakrar R, Vietti T, Chen M, Cohen J, Siniakowicz K, Qi M, McGarrigle J, Graham AC, Lyle S, Harlan DM, Greiner DL, Oberholzer J, Weir GC, Langer R, Anderson DG. Combinatorial hydrogel library enables identification of materials that mitigate the foreign body response in primates. Nat Biotechnol 2016;34:345–52. 26807527 10.1038/nbt.3462 PMC4904301

[168] Wu J , Chen A, Zhou Y, Zheng S, Yang Y, An Y, Xu K, He H, Kang J, Luckanagul JA, Xian M, Xiao J, Wang Q. Novel H2S-releasing hydrogel for wound repair via in situ polarization of M2 macrophages. Biomaterials 2019;222:119398. 31487582 10.1016/j.biomaterials.2019.119398

[169] Kim BS , Kim SH, Kim K, An YH, So KH, Kim BG, Hwang NS. Enzyme-mediated one-pot synthesis of hydrogel with the polyphenol cross-linker for skin regeneration. Mater Today Bio 2020;8:100079. 10.1016/j.mtbio.2020.100079 PMC7575804 33103105

[170] Leach DG , Dharmaraj N, Piotrowski SL, Lopez-Silva TL, Lei YL, Sikora AG, Young S, Hartgerink JD. STINGel: controlled release of a cyclic dinucleotide for enhanced cancer immunotherapy. Biomaterials 2018;163:67–75. 29454236 10.1016/j.biomaterials.2018.01.035 PMC5840037

[171] Wang K , Dong R, Tang J, Li H, Dang J, Zhang Z, Yu Z, Guo B, Yi C. Exosomes laden self-healing injectable hydrogel enhances diabetic wound healing via regulating macrophage polarization to accelerate angiogenesis. Chem Eng J 2022;430:132664.

[172] Krieger J , Ogle M, McFaline-Figueroa J, Segar CE, Temenoff JS, Botchwey EA. Spatially localized recruitment of anti-inflammatory monocytes by SDF-1α-releasing hydrogels enhances microvascular network remodeling. Biomaterials 2016;77:280–90. 26613543 10.1016/j.biomaterials.2015.10.045 PMC4698334

[173] Lohmann N , Schirmer L, Atallah P, Wandel E, Ferrer RA, Werner C, Simon JC, Franz S, Freudenberg U. Glycosaminoglycan-based hydrogels capture inflammatory chemokines and rescue defective wound healing in mice. Sci Transl Med 2017;9:eaai9044. 28424334 10.1126/scitranslmed.aai9044

[174] Gajanayake T , Olariu R, Leclère FM, Dhayani A, Yang Z, Bongoni AK, Banz Y, Constantinescu MA, Karp JM, Vemula PK, Rieben R, Vogelin E. A single localized dose of enzyme-responsive hydrogel improves long-term survival of a vascularized composite allograft. Sci Transl Med 2014;6:249ra110. 10.1126/scitranslmed.3008778 25122638

[175] Li Y , Chen X, Jin R, Chen L, Dang M, Cao H, Dong Y, Cai B, Bai G, Gooding JJ, Liu S, Zou D, Zhang Z, Yang C. Injectable hydrogel with MSNs/microRNA-21-5p delivery enables both immunomodification and enhanced angiogenesis for myocardial infarction therapy in pigs. Sci Adv 2021;7:eabd6740. 33627421 10.1126/sciadv.abd6740 PMC7904259

[176] Wang C , Wang J, Zhang X, Yu S, Wen D, Hu Q, Ye Y, Bomba H, Hu X, Liu Z, Dotti G, Gu Z. In situ formed reactive oxygen species–responsive scaffold with gemcitabine and checkpoint inhibitor for combination therapy. Sci Transl Med 2018;10:eaan3682. 29467299 10.1126/scitranslmed.aan3682

[177] Eelkema R , Pich A. Pros and Cons: supramolecular or macromolecular: what is best for functional hydrogels with advanced properties? Adv Mater 2020;32:1906012. 10.1002/adma.201906012 31919957

[178] Chaudhuri O. Viscoelastic hydrogels for 3D cell culture. Biomater Sci 2017;5:1480–90. 28584885 10.1039/c7bm00261k

[179] Chaudhuri O , Gu L, Klumpers D, Darnell M, Bencherif SA, Weaver JC, Huebsch N, Lee H, Lippens E, Duda GN, Mooney DJ. Hydrogels with tunable stress relaxation regulate stem cell fate and activity. Nat Mater 2016;15:326–34. 26618884 10.1038/nmat4489 PMC4767627

[180] Lee H-p , Gu L, Mooney DJ, Levenston ME, Chaudhuri O. Mechanical confinement regulates cartilage matrix formation by chondrocytes. Nat Mater 2017;16:1243–51. 28967913 10.1038/nmat4993 PMC5701824

[181] McKinnon DD , Domaille DW, Cha JN, Anseth KS. Biophysically defined and cytocompatible covalently adaptable networks as viscoelastic 3D cell culture systems. Adv Mater 2014;26:865–72. 24127293 10.1002/adma.201303680 PMC4582033

[182] Ansari S , Chen C, Hasani-Sadrabadi MM, Yu B, Zadeh HH, Wu BM, Moshaverinia A. Hydrogel elasticity and microarchitecture regulate dental-derived mesenchymal stem cell-host immune system cross-talk. Acta Biomater 2017;60:181–9. 28711686 10.1016/j.actbio.2017.07.017 PMC5581234

[183] Ji Y , Li J, Wei Y, Gao W, Fu X, Wang Y. Substrate stiffness affects the immunosuppressive and trophic function of hMSCs via modulating cytoskeletal polymerization and tension. Biomater Sci 2019;7:5292–300. 31612176 10.1039/c9bm01202h

[184] Fukuyama Y , Yuki Y, Katakai Y, Harada N, Takahashi H, Takeda S, Mejima M, Joo S, Kurokawa S, Sawada S, Shibata H, Park EJ, Fujihashi K, Briles DE, Yasutomi Y, Tsukada H, Akiyoshi K, Kiyono H. Nanogel-based pneumococcal surface protein A nasal vaccine induces microRNA-associated Th17 cell responses with neutralizing antibodies against Streptococcus pneumoniae in macaques. Mucosal Immunol 2015;8:1144–53. 25669148 10.1038/mi.2015.5 PMC4762909

[185] Rostam HM , Fisher LE, Hook AL, Burroughs L, Luckett JC, Figueredo GP, Mbadugha C, Teo ACK, Latif A, Kämmerling L, Day M, Lawler K, Barrett D, Elsheikh S, Ilyas M, Winkler DA, Alexander MR, Ghaemmaghami AM. Immune-instructive polymers control macrophage phenotype and modulate the foreign body response in vivo. Matter 2020;2:1564–81.

[186] Lee J , Jeon O, Kong M, Abdeen AA, Shin JY, Lee HN, Lee YB, Sun W, Bandaru P, ALT DS, Lee K, Kim HJ, Lee SJ, Chaterji S, Shin SR, Alsberg E, Khademhosseini A. Combinatorial screening of biochemical and physical signals for phenotypic regulation of stem cell–based cartilage tissue engineering. Sci Adv 2020;6:eaaz5913. 32494742 10.1126/sciadv.aaz5913 PMC7244269

[187] Xue J , Schmidt SV, Sander J, Draffehn A, Krebs W, Quester I, Nardo DD, Gohel TD, Emde M, Schmidleithner L, Ganesan H, Nino-Castro A, Mallmann MR, Labzin L, Theis H, Kraut M, Beyer M, Latz E, Freeman TC, Ulas T, Schultze JL. Transcriptome-based network analysis reveals a spectrum model of human macrophage activation. Immunity 2014;40:274–88. 24530056 10.1016/j.immuni.2014.01.006 PMC3991396

[188] Bartneck M , Heffels K-H, Pan Y, Bovi M, Zwadlo-Klarwasser G, Groll J. Inducing healing-like human primary macrophage phenotypes by 3D hydrogel coated nanofibres. Biomaterials 2012;33:4136–46. 22417617 10.1016/j.biomaterials.2012.02.050

[189] Abuawad A , Mbadugha C, Ghaemmaghami AM, Kim DH. Metabolic characterisation of THP-1 macrophage polarisation using LC–MS-based metabolite profiling. Metabolomics 2020;16:1–14. 10.1007/s11306-020-01656-4 PMC7049298 32114632

